# *C*-Diazeniumdiolate Graminine in the Siderophore Gramibactin Is Photoreactive and Originates from Arginine

**DOI:** 10.1021/acschembio.2c00593

**Published:** 2022-11-10

**Authors:** Christina Makris, Jeffrey R. Carmichael, Hongjun Zhou, Alison Butler

**Affiliations:** Department of Chemistry & Biochemistry, University of California, Santa Barbara, California 93106-9510, United States

## Abstract

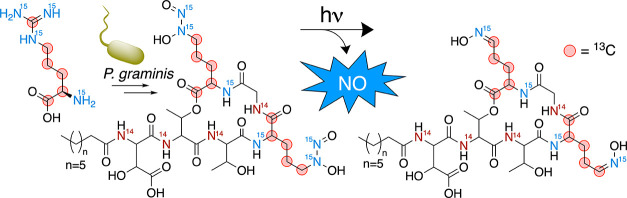

Siderophores are synthesized by microbes to facilitate iron acquisition required for growth. Catecholate, hydroxamate, and α-hydroxycarboxylate groups comprise well-established ligands coordinating Fe(III) in siderophores. Recently, a *C*-type diazeniumdiolate ligand in the newly identified amino acid graminine (Gra) was found in the siderophore gramibactin (Gbt) produced by *Paraburkholderia graminis* DSM 17151. The N–N bond in the diazeniumdiolate is a distinguishing feature of Gra, yet the origin and reactivity of this *C*-type diazeniumdiolate group has remained elusive until now. Here, we identify l-arginine as the direct precursor to l-Gra through the isotopic labeling of l-Arg, l-ornithine, and l-citrulline. Furthermore, these isotopic labeling studies establish that the N–N bond in Gra must be formed between the *N*^δ^ and *N*^ω^ of the guanidinium group in l-Arg. We also show the diazeniumdiolate groups in apo-Gbt are photoreactive, with loss of nitric oxide (NO) and H^+^ from each d-Gra yielding *E*/*Z* oxime isomers in the photoproduct. With the loss of Gbt’s ability to chelate Fe(III) upon exposure to UV light, our results hint at this siderophore playing a larger ecological role. Not only are NO and oximes important in plant biology for communication and defense, but so too are NO-releasing compounds and oximes attractive in medicinal applications.

## Introduction

**Figure 1:**
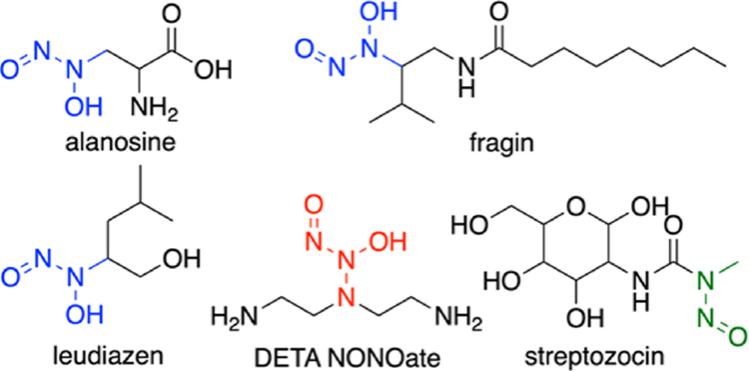
Selected N–N bonded compounds. *C*-diazeniumdiolate in alanosine, fragin, and leudiazen are shown in blue, the *N*-diazeniumdiolate in DETA NONOate is shown in red, and the *N-*nitrosourea in streptozocin is shown in green.

**Figure 2:**
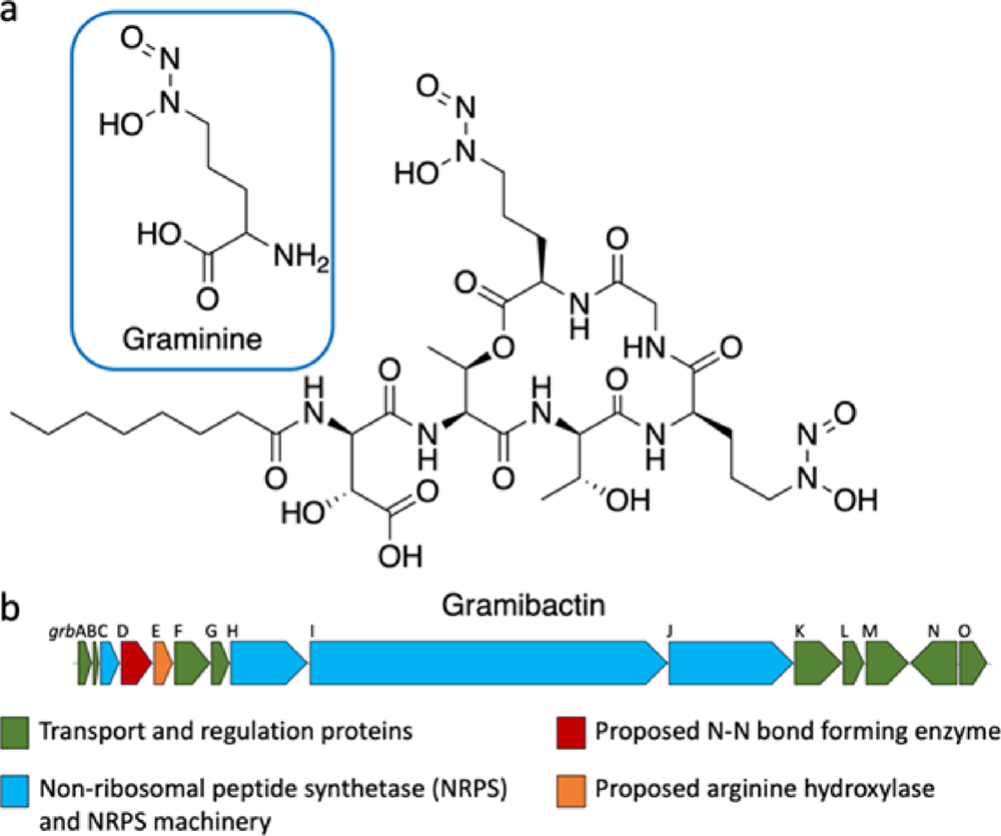
(a) Structure of Gbt with Gra in inset; (b) BGC of Gbt.

A disproportionate sum of pharmaceuticals incorporate N–N bonds. Pharmaceuticals are often inspired by natural products, with at least 200 reported with an N–N bond.^1,2^ From this subset of natural products, only a few harbor a *C*-type diazeniumdiolate functional group (Figure 1).^2^*C*-type diazeniumdiolates, like alanosine,^3,4^ fragin,^5,6^ and leudiazen,^7^ are defined by the nitrosohydroxylamine group bonded to a carbon as opposed to *N*-type diazeniumdiolates, such as (Z)-1-[N-(2-aminoethyl)-N-(2-ammonioethyl)amino]diazen-1-ium-1,2-diolate (DETA NONOate)^8^ in which the nitrosohydroxylamine group is appended to a nitrogen atom (Figure 1). In 2018, the siderophore gramibactin (Gbt) was reported from *Paraburkholderia graminis* DSM 17151 with a new *C*-diazeniumdiolate amino acid, graminine (Gra; Figure 2).^9,10^ Siderophores are small molecules produced by bacteria to facilitate the acquisition of iron, which is essential for microbial growth.^11^ Gbt is comprised of six amino acids, which includes two d-Gra residues, along with Gly, d-*allo* Thr, l-Thr, and d-*threo* β-hydroxyAsp residues of which each Gra and β-hydroxyaspartic acid (β-OH-Asp) coordinate Fe(III).^12^

The biological signaling properties of nitric oxide (NO) are widespread in nature. Diazeniumdiolates, which are recognized as potential NO-donor compounds, are classified as C-, N-, O-, or S-type compounds among which the reactivity of NONOates has received the most attention. A particularly attractive feature of NONOates, which have potential clinical applications,^13−15^ is their photoreactivity, producing two equivalents of NO. In contrast to NONOates, which have yet to be identified in natural products, little is known about the reactivity of *C*-type diazeniumdiolate natural products and the conditions under which NO may be released (Figure 1).^8,16^

The origin and biosynthesis of the N–N bond in natural products have attracted much interest,^1,17^ in part due to the anticancer drug streptozocin (Zanosar, Figure 1) isolated from *Streptomyces achromogenes* var. *streptozoticus* NRRL 2697, which is effective against pancreatic cancers.^18,19^ The enzyme SznF in the biosynthetic gene cluster (BGC) of streptozocin is reported to catalyze the formation of the *N-*nitrosourea group (Figure 1).^19^ Prior to N–N bond formation, the heme-dioxygenase-like domain of SznF hydroxylates *N*^ω^-methyl-l-Arg at both the guanidinium *N*^δ^ and the unmethylated *N*^ω^ positions.^19−21^ Following hydroxylation, the cupin domain of SznF is proposed to catalyze the oxidative rearrangement of *N*^δ^-hydroxy-*N*^ω^-hydroxy-*N*^ω^-methyl-l-Arg forming the N–N bond of streptozocin; however, the mechanism of N–N bond formation remains elusive.^19^

In the BGC for Gbt (Figure 2), the enzymes GrbE and GrbD have been identified through gene deletion studies to be essential for the formation of Gra.^10^ GrbE shares sequence homology to several reported arginine hydroxylases, including AglA/AlpD and Mhr24, which are *N*^δ^-hydroxylases,^22−24^ and DcsA, which is a *N*^ω^-hydroxylase (Figure S1).^25^ GrbD shares sequence homology to only the cupin domain in SznF (Figure S1).^19^ The homologies of GrbE to the Arg hydroxylases and GrbD to one domain of SznF is suggestive of l-Arg as the precursor of l-Gra, yet direct experiments to investigate l-Arg and the source of l-Gra have not been reported.

Herein, we report that the Gra *C*-diazeniumdiolate groups in apo-Gbt are photoreactive, losing an equivalent of NO and H^+^ from each d-Gra residue. Through isotopic feeding studies, we demonstrate that Gra in Gbt originates from Arg. We further structurally characterize the Gbt photoproduct as a mixture of *E*/*Z* oxime isomers.

## Results and Discussion

### Apo-Gbt Loses Two Equivalents of Nitric Oxide upon Photolysis

**Figure 3:**
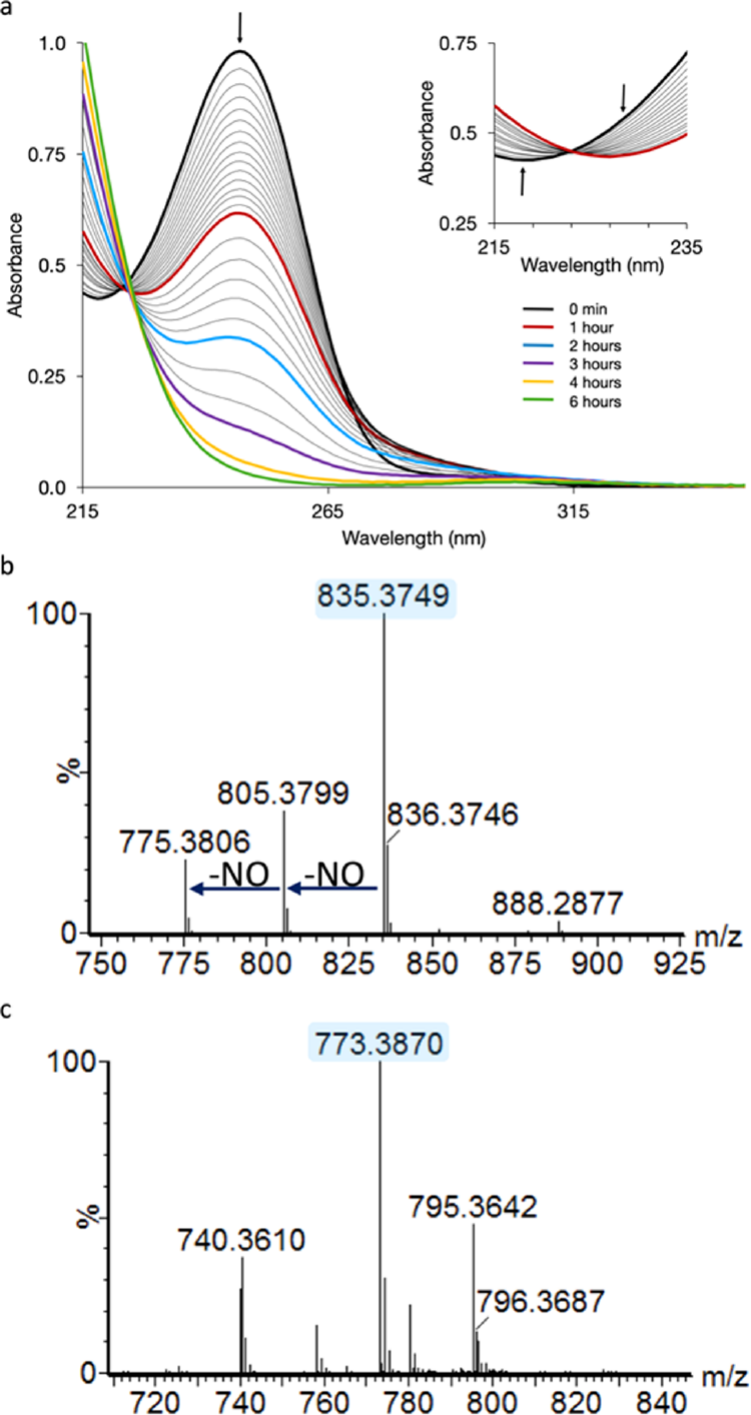
UV photolysis of apo-Gbt. (a) UV-absorption spectra of apo-Gbt (44 μM) as a function of the time of photolysis (253.7 nm bandpass filter; 0–6 h in 5 mM MOPS pH 8.0). From 0 to 1 h, spectra were obtained at 4 min intervals; from 1 to 2 h, spectra were obtained at 10 min intervals; and from 2 to 3 h, spectra were obtained at 20 min intervals. Inset showing the isosbestic point between time 0 and 1 h; (b) MS of apo-Gbt; (c) MS of the photoproduct of Gbt.

Upon irradiation of apo-Gbt (5 mM, 3-(N-morpholino)propane sulfonic acid (MOPS), pH 8) with UV light (e.g., λ 254 nm Hg(Ar) pen lamp and in sunlight), the characteristic pH 8 diazeniumdiolate absorption band at 248 nm disappears (Figure 3a). Mass spectrometric analysis of apo-Gbt, *m/z* 835.3 [M + H]^+^, shows two successive mass losses of 30, as well as a mass at *m/z* 888.3 [M – 2H + Fe]^+^ indicative of the Fe(III)-Gbt complex (Figure 3b). The observed mass losses of *m/z* 30 in the mass spectrum are characteristic of diazeniumdiolates, reflecting the lability of the N–N bond in the presence of ionization energy in the mass spectrometer.^10^

Analysis of the purified photoproduct by mass spectrometry shows a new mass at *m/z* 773.3 [M + H]^+^ (Figure 3c). The difference of 62 mass units from apo-Gbt at *m/z* 835.3 [M + H]^+^ is consistent with the loss of two equivalents each of NO and H^+^. An Fe(III) bound mass was not observed in the mass spectrometry (MS) of the photoproduct, indicating a change in the Fe(III) coordinating groups. The mass of the photoproduct of ^15^N-enriched Gbt shows a mass loss of *m/z* 64, consistent with the release of two equiv. each of ^15^NO and H^+^. To investigate the biological relevance of the photoreactivity, aliquots were taken directly from an actively growing culture of *P. graminis* DSM 17151, two of which were photolyzed with different UV light sources. One aliquot was irradiated with a Hg(Ar) pen lamp and the other with sunlight. Following irradiation, the cells were pelleted and the crude supernatant was analyzed by ultra-high performance liquid chromatography (UPLC-MS), revealing only the photoproduct *m/z* 773.3 [M + H]^+^ without any trace of apo-Gbt, *m/z* 835 (Figure S2). In contrast, an aliquot from the same culture which was not exposed to UV light did not contain any of the photoproduct (Figure S2). The results of the colorimetric Griess test, which detects nitrite,^26−28^ are also consistent with the photoinduced release of NO with subsequent oxidation to NO_2_^**–**^ (Figure S3). Thus, the UV and mass spectral changes, along with the Griess test results suggest the photoreaction leads to the loss of NO and a H^+^ from each of the two d-Gra residues in apo-Gbt.

The β-hydroxyaspartate coordinated to Fe(III) in Gbt is photoreactive, complicating the investigation of possible NO labilization while bound to Fe(III).^29,30^ Analysis of the Fe(III)-Gbt photoproducts by UPLC-MS initially shows the molecular ion *m/z* 842 [M – 2H + Fe]^+^, corresponding to the loss of CO_2_ and two H^+^’s from the β-OH-Asp, but retaining the coordination to iron with the two diazeniumdiolate ligands (Figure S4). Initially, NO is not labilized in the photolysis of Fe(III)-Gbt; however, further mechanistic investigations of the photoreactivity are underway.

The λ_max_ of the diazeniumdiolate in apo-Gbt is strongly pH dependent, shifting from 248 nm at pH 10 to 220 nm at pH 2.^9^ Not surprisingly, we find the attendant photoreactivity of apo-Gbt at low pH, as measured by the disappearance of the 220 nm absorption peak, is reduced both on irradiation at 254 nm and in sunlight. The reduced photosensitivity at low pH reflects the poor overlap of the irradiating wavelength and blue-shift of the absorption band at pH 2 (λ_max_ 220 nm) compared to pH 8 (λ_max_ 248 nm).

### Isotopic Labeling Establishes Gra Originates from Arg

**Figure 4:**
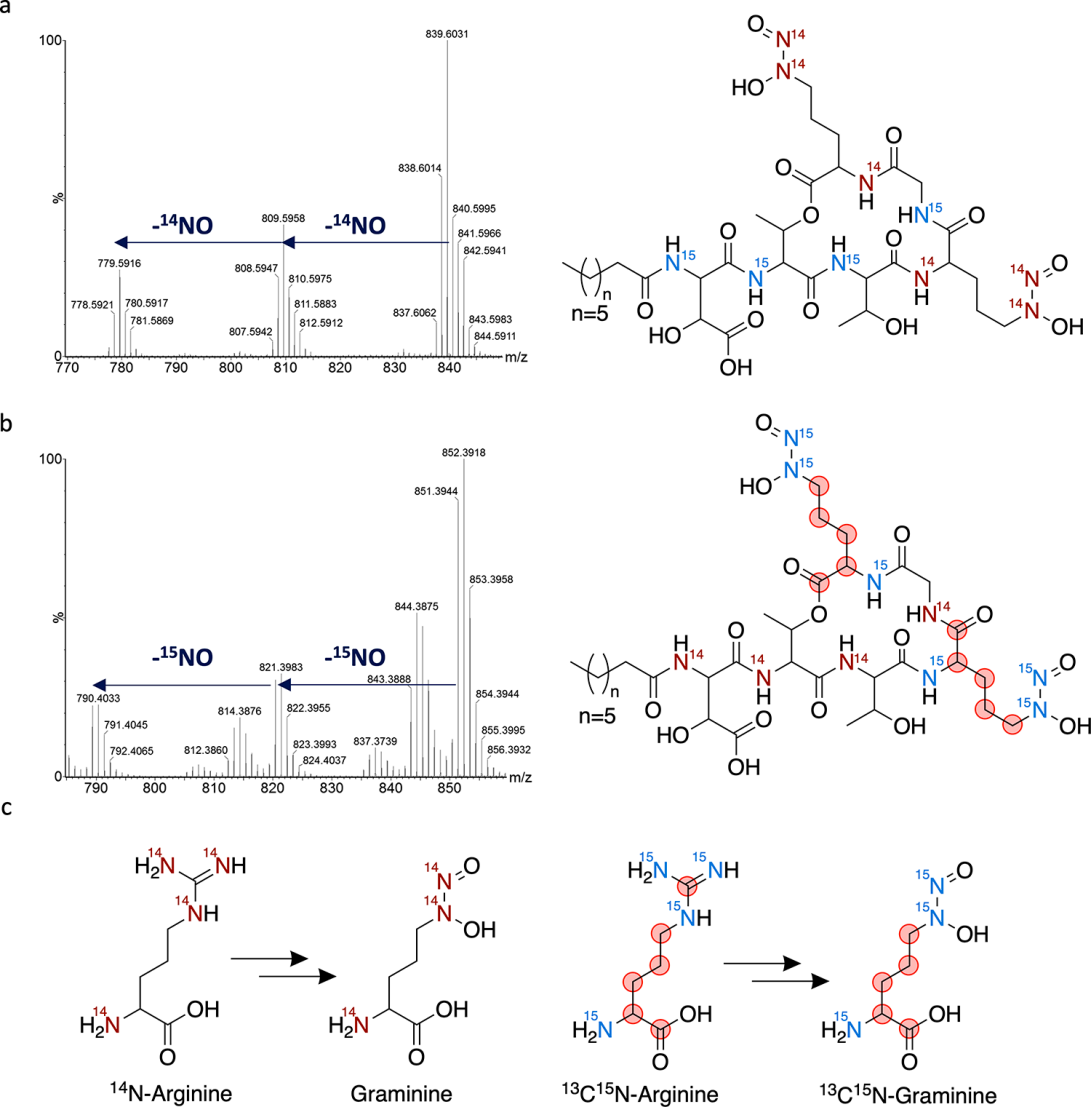
Isotope feeding studies demonstrate l-Gra originates from l-Arg. (a) Gbt isolated from the *P. graminis* DSM 17151 culture grown on ^15^NH_4_Cl and ^14^N-Arg. Isotopic mass of Gbt at *m/z* 839 is present along with two mass losses of ^14^NO; (b) Gbt isolated from the *P. graminis* DSM 17151 culture grown on ^14^NH_4_Cl and ^13^C_6_^15^N_4_-Arg. Isotopic mass of Gbt at *m/z* 851 is present along with two mass losses of ^15^NO; (c) Scheme depicting isotopic labeling of Gra originating from ^14^N-Arg or from ^13^C_6_^15^N_4_-Arg.

To investigate the origin of Gra, we employed ^13^C and ^15^N isotopic feeding studies in the growth of *P. graminis* DSM 17151. When this strain is grown with ^15^NH_4_Cl as the sole nitrogen source, an isotopic mass for Gbt of M + 10 is observed (i.e., *m/z* 845.3 [M + H]^+^), consistent with ^15^N-enrichment at each nitrogen (Figure S5). However, when *P. graminis* DSM 17151 is cultured with both ^15^NH_4_Cl and ^14^N-l-Arg, an isotopic mass at *m/z* 839.3 [M + H]^+^ is observed for Gbt which is four mass units higher than that for Gbt (Figures 4 and S6). The isotopic mass of M + 4 is consistent with ^15^N incorporation into d-*threo* OH-Asp, l-Thr, d-*allo* Thr, and Gly, whereas the nitrogens in Gra are consistent with incorporation from the supplemented ^14^N-l-Arg (Figure 4c). MS analysis of the M + 4 Gbt derivative substantiates the incorporation of ^14^N in each Gra residue with two ion mass fragments of 30 (^14^NO) rather than 31 (^15^NO) (Figure 4a). Upon photolysis of the partially ^15^N-enriched apo-Gbt, a photoproduct with a mass of *m/z* 777.3 [M + H]^+^ is observed (Figures S6 and S7), also consistent with the incorporation of ^14^N Gra in the M + 4 Gbt derivative. While photolysis of fully ^15^N enriched Gbt leads to a photoproduct with a mass loss of 64, we observe a mass loss of 62 in this M + 4 Gbt derivative, consistent with ^14^N Gra (Figures S5 and S7).

To further probe the origin of Gra in Gbt, the *P. graminis* DSM 17151 culture medium was supplemented with 1 mM ^13^C^15^N-l-Arg. The resulting Gbt showed isotopic masses at *m/z* 851.3 (M + 16) and 852.3 (M + 17; Figure 4b), as well as 853.3 (M + 18). The M + 16 mass is expected based on the isotopic labeling of five ^13^C’s and three ^15^N’s per Gra (Figure 4c). The M + 17 and M + 18 Gbt likely result from the incorporation of an ^15^N released from ^13^C^15^N-l-Arg and then taken up in the biosynthesis of the other amino acids, as confirmed by nuclear magnetic resonance (NMR) (Figures S8–S20).

NMR characterization of Gbt purified from *P. graminis* DSM 17151 grown in the presence of ^13^C^15^N-Arg and dissolved in hexadeuterodimethyl sulfoxide (DMSO-d_6_) and D_2_O (^1^H, ^13^C, heteronuclear single quantum coherence-distortionless enhancement by polarization transfer (HSQC-DEPT), correlated spectroscopy (COSY), ^1^H–^13^C heteronuclear multiple bond correlation (HMBC), ^13^C–^13^C COSY, ^1^H–^15^N HMBC, and ^13^C incredible natural abundance double quantum transfer experiment (INADEQUATE)) confirms the incorporation of fully labeled ^13^C^15^N Gra residues in Gbt (Figures S8–S20). Connectivity of ^13^C-enriched Cα, Cβ, Cγ, and Cδ in each Gra residue was obtained by ^13^C INADEQUATE and ^13^C–^13^C COSY experiments (Figures S17 and S18). ^1^H NMR spectra show mixtures of ^14^N–H and ^15^N–^1^H coupled (*J* = 90 Hz) amides at each amino acid residue (Figure S10).^31^ The ^15^N-labeling in Gly, each Thr and β-OH-Asp must result from ^15^NH_3_ released from ^13^C^15^N-Arg, either as a byproduct of arginase and the urea cycle,^32−36^ or during the putative oxidative conversion of hydroxy-Arg to Gra, and the subsequent incorporation of ^15^NH_3_ in the biosynthesis of these amino acids. The M + 17 Gbt isotopic mass is consistent with the incorporation of one ^15^N-labeled amino acid at any of the four amino acids distinct from Gra (Figures S10 and S21). A less intense M + 18 isotopic mass is also observed (Figure 4b) suggestive of the incorporation of two ^15^NH_3_-labeled amino acids. Incorporation of ^13^C released by these processes is not detected in the isolated Gbt reflecting the higher concentration of other carbon sources in the growth medium.

It has been speculated that Gra originates from l-ornithine;^17^ therefore we also investigated whether ^14^N-ornithine and ^14^N-citrulline added to the *P. graminis* DSM 17151 growth medium with ^15^NH_4_Cl are converted to Gra. l-Orn and l-Cit are both on the microbial biosynthetic route to l-Arg,^32−34^ and therefore it may be more energetically favorable for the microbe to use the added ^14^N-l-Orn or ^14^N-l-Cit before initiating the de novo biosynthesis of l-Arg using the added ^15^NH_4_Cl (Figure S21). Gbt isolated from cultures supplemented with ^14^N-l-Orn show two primary isotopic masses of Gbt, *m/z* 841.3 [M + H]^+^ (i.e., M + 6) and *m/z* 843.3 [M + H]^+^ (i.e., M + 8), with attendant mass losses of ^15^NO present in the mass spectrum (Figure S22). ^14^N-l-Orn will yield l-Arg with both guanidinium ^15^*N*^ω^’s enriched. Both the peptidyl amide nitrogen and hydroxylamine nitrogen in the resulting Gra remain as naturally abundant ^14^N, while the nitroso nitrogen is ^15^N-enriched (Figure S22). In contrast to ^14^N-l-Orn, ^14^N-l-Cit would be expected to yield l-Arg with the two guanidium *N*^ω^’s existing as an equivalent mixture of ^14^N and ^15^N (Figure S23). When cultures were supplemented with ^14^N-l-Cit, a range of isotopic masses are observed for Gbt (Figure S23), consistent with Gra arising from distinct isotopically enriched l-Arg residues. The results of isotopic feeding of *P. graminis* DSM 17151 with ^14^N-l-Orn and ^14^N-l-Cit in the presence of ^15^NH_4_Cl are thus consistent with l-Gra originating from l-Arg and suggest the conversion is enzymatic. The observation of two distinct isotopic molecular ions resulting from ^14^N-l-Orn supplementation in contrast to an array of isotopic species with ^14^N-l-Cit supplementation demonstrates that the N–N bond must form between the ^14^*N*^δ^ of l-Arg, and either of the ^15^*N*^ω^ in the guanidinium groups. Investigations into the mechanism of the oxidative conversion of Arg to Gra are in progress.

### Photolysis of Isotopically Labeled Gbt Reveals the Photoproduct Is an Oxime

**Figure 5:**
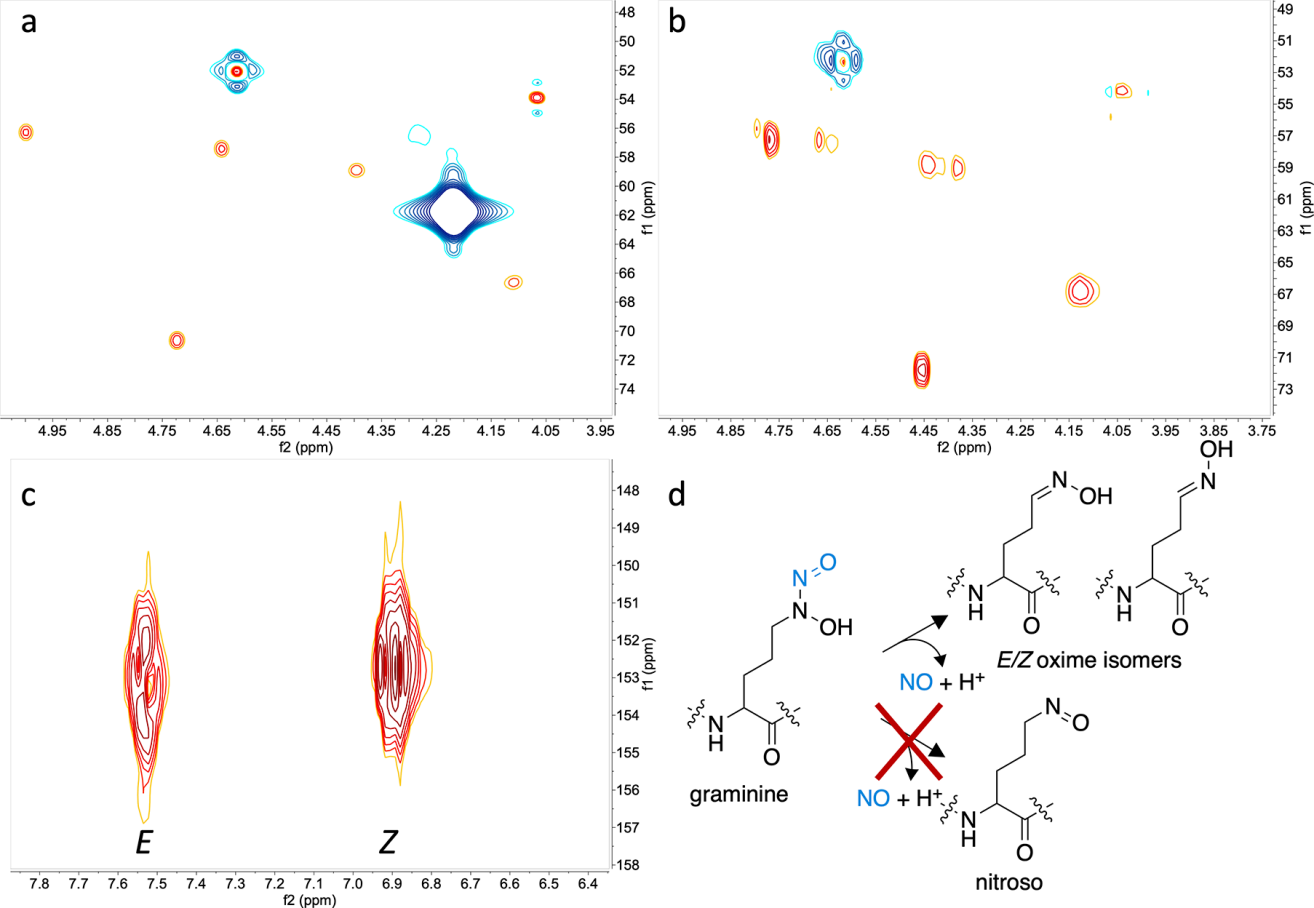
Multiplicity edited ^1^H–^13^C HSQC NMR spectra show the formation of *E* and *Z* oxime isomers upon photolysis of ^13^C^15^N-Gra-enriched-Gbt (pD 8, 6.2 mM, *P_i_* in 99.9% D_2_O). (a) ^1^H–^13^Cδ correlations in Gra residues of apo-Gbt at 4.20, 61.66 ppm and 4.22, 61.93 ppm; (b) region of HSQC showing disappearance of the ^1^H–^13^Cδ correlations in the photoproduct; (c) new downfield ^1^H–^13^C HSQC correlations in photolyzed ^13^C^15^N-Gra-enriched Gbt, with chemical shifts consistent with *E*/*Z* oxime isomers; (d) scheme showing release of NO from Gra yields *E* and *Z* oxime isomers but not a nitroso photoproduct. Spectra collected in D_2_O.

The ^13^C^15^N-isotopically enriched Gra residues in Gbt were also used to characterize the photoproduct of apo-Gbt, which could produce nitroso or oxime products (Figure 5). Immediately following the complete photolysis of apo-Gbt in a quartz NMR tube (pD 8.0, 6.2 mM phosphate buffer, 99.9% D_2_O), as indicated by the disappearance of the diazeniumdiolate absorption band at 248 nm, the reaction solution was evaluated by NMR (Figures 5 and S24–S29). The ^1^H (^13^C and ^15^N decoupled) and ^13^C NMR spectra show two new ^1^H resonances at 6.90 and 7.53 ppm and two new ^13^C resonances at 152.72 and 153.10 ppm not present in Gbt (Figures 5c and S24–S26). The ^1^H-^13^C HSQC spectrum of the photoproduct shows that the two new ^1^H resonances correlate to the two new ^13^C resonances (Figure 5c). The formation of an oxime is consistent with the Cδ of the Gra residues undergoing a change from sp^3^ to sp^2^ hybridization. Additionally, both *E* and *Z* oxime isomers are observed for both Gra residues, with the *E* isomer proton being shifted further downfield than the *Z* configuration.^37,38^ The Cδ ^1^H and ^13^C shifts of both Gra residues in apo-Gbt (Figure 5a) disappear upon photolysis (Figure 5b). Coupling between ^15^N, ^13^C, and ^1^H nuclei on the ^13^C^15^N-enriched-Gra residues causes inconsistencies in the phasing, as can be observed at the Cα’s of the Gra residues, which are adjacent to a ^15^N and two ^13^C nuclei (Figures S11 and S16). HSQC resonances from the remaining amino acid residues in Gbt derive from the natural abundance of ^13^C. In comparison, the ^1^H–^13^C correlations of Cα, Cβ, Cγ, and Cδ in ^13^C-enriched Gra are apparent, including the disappearance of the ^1^H–^13^C correlation from each Gra Cδ in the photoproduct (Figure 5a,b). Full NMR characterization of the photoproduct shows the rest of the structure remains unchanged. A TOtal Correlated SpectroscopY (TOCSY) NMR experiment establishes that the *E* and *Z* oxime conformations are present in the complete ^1^H spin systems of both Gra1 and Gra2 photoproducts (Figure S30). Thus, the NMR results establish the photoproduct of apo-Gbt is a mixture of *E*/*Z* oxime isomers (Figure 5d). The ratio of the ^1^H resonance integrations of the *E* to *Z* oxime (1:1.7) immediately after photolysis shows the *Z* configuration is the dominant form. After 2 days at −20 °C, the peak integration (1:0.7) shows a shift to the E configuration, which has been reported as the more biologically active isomer.^39^ The *E*/*Z* isomerization process is under further investigation.

## Conclusions

**Figure 6:**
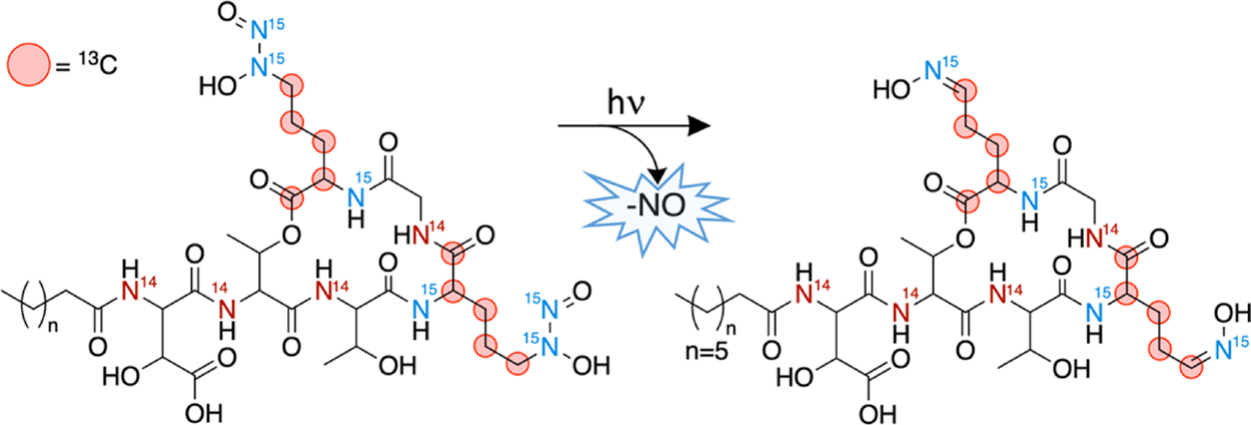
Photolysis of ^13^C^15^N-Gra-enriched-Gbt yields *E*/*Z* oxime isomers.

Apo-Gbt is photoreactive, losing NO and H^+^ from each d-Gra upon irradiation with UV light (Figure 6). The downfield shift of the Gra Cδ ^1^H and ^13^C resonances in the ^13^C^15^N-enriched Gbt photoproduct is consistent with the formation of an oxime. The 0.6 ppm difference between the new ^1^H resonances (6.90, 7.53 ppm) is indicative of a mixture of *E*/*Z* oxime isomers.^37^ The mass loss of 64 observed in the ^13^C^15^N-Gbt photoproduct is consistent with release of ^15^NO and H^+^ from each Gra. Thus, the photoreactivity of this *C*-diazeniumdiolate natural product is established, and is among the first examples of photorelease of NO from a *C*-diazeniumdiolate. Isotopic feeding of *P. graminis* DSM 17151 with ^13^C_6_^15^N_4_l-Arg establishes that it is the precursor to l-Gra, with all ^13^C’s and ^15^N’s in Gra originating from the enriched-Arg. Moreover, we demonstrate with isotopic labeling that l-Orn and l-Cit are not direct precursors to l-Gra, with these results demonstrating that the N–N bond is formed between *N*^δ^ and *N*^ω^ of the l-Arg guanidinium group. In ongoing research, we are investigating the role of GrbE in l-Arg hydroxylation and the role of GrbD in the oxidative rearrangement of hydroxy-arginine in the formation of the N–N bond in l-Gra.

Oximes are an established class of pharmaceuticals, with several FDA-approved oxime-based therapeutics used as antidotes for organophosphate poisoning, as well as in cephalosporin antibiotics.^40^ In addition, oximes and NO are biologically active in plants, serving functions facilitating plant defense, communication, and growth.^39,41,42^ While the pathways involved in NO signaling in response to stress are still largely unknown, NO has been shown to regulate gene expression and hormonal activity, as well as provide protection under oxidative stress by interacting with reactive oxygen species.^42^ It is plausible that the photochemical release of NO and the formation of the oxime could be an unintended side reaction of Gbt, given oximes derived from amino acids commonly serve as intermediates to defensive compounds and plant hormones.^39^ Free Gra could play a role in plant biology as a unique photoreactive precursor to produce an oxime and release NO when exposed to sunlight. Given that the *C*-diazeniumdiolate siderophores currently reported in the literature are all isolated from rhizospheric bacteria, Gbt may serve the dual purpose of Fe(III) acquisition, as well as providing a source of NO upon exposure to sunlight as part of a symbiotic relationship with the associated root nodules.^10^

## Methods

### General Experimental Procedures

UV–Visible spectra were obtained on an Agilent Technologies Cary 300 UV–vis spectrometer. NMR spectroscopy was carried out at 25 °C on a Bruker 500 MHz spectrometer equipped with a Prodigy cold probe (^1^H, ^13^C, ^1^H–^13^C multiplicity edited HSQC, ^1^H–^1^H COSY, ^13^C–^13^C COSY, ^1^H–^13^C HMBC, ^1^H–^15^N HMBC, ^13^C INADEQUATE, and TOCSY) or on a Varian Inova 600 MHz spectrometer equipped with a ^1^H, ^13^C, ^15^N triple resonance inverse detection probe (^1^H with or without ^13^C, ^15^N, or ^13^C/^15^N decoupling, and ^1^H–^15^N HMBC with or without ^13^C decoupling). Spectra were collected in DMSO-d_6_ or D_2_O. Spectra were indirectly referenced by the residual solvent peak or ^2^H lock. MS analysis was carried out on a Waters Xevo G2-XS QTof with positive mode electrospray ionization coupled to an AQUITY UPLC H-Class system with a Waters BEH C18 column. Gbt samples were run with a linear gradient of 0 to 100% acetonitrile (0.1% formic acid) in ddH_2_O (0.1% formic acid) over 10 min. Deionized water was dispensed from a Milli-Q IQ 7000 water purification system (Resistivity 18.2 MΩ). All glassware was acid-washed with 4 M HCl and subsequently rinsed with Milli-Q water.

### Bacterial Growth and Siderophore Isolation

*P. graminis* DSM 17151 was obtained from the German collection of Microorganisms and Cell Cultures (Deutsche Sammlung von Mikroorganismen und Zellkulturen, DSMZ). *P. graminis* DSM 17151 was maintained on Luria–Bertani (LB) agar plates at 30 °C. Single colonies were inoculated in 3 mL of LB media and grown for 24 h shaking at 30 °C, 180 rpm. The 3 mL starter cultures were used to inoculate 1 L cultures of iron-depleted MM9 medium (7 g K_2_HPO_4_, 2 g KH_2_PO_4_, 0.59 g NaCl, 1 g NH_4_Cl, 0.1 g MgSO_4_, and 5 g disodium succinate amended with 20 mL steri-filtered 50% (w/v) glucose following autoclaving for 20 min at 121 °C)^9^ in a 2 L Fernbach flask. Cultures were grown at 30 °C on an orbital shaker at 180 rpm. Microbial growth was monitored by OD_600nm_ and cultures were harvested when growth reached late log phase with a positive Fe(III)-chrome azurol assay response.^43^ Culture supernatants were obtained by centrifugation at 6000 rpm for 30 min at 4 °C (SLA-3000 rotor, Thermo Scientific). To extract Gbt, the culture supernatant was decanted and shaken with 100 g Amberlite® XAD-4 resin. The XAD-4 resin was prepared by washing with methanol and then equilibrating with Milli-Q water. The resin and supernatant were allowed to equilibrate for 4 h at 120 rpm. The resin was filtered from the supernatant and washed with 0.5 L Milli-Q water. The adsorbed organics were eluted with 80% aq. methanol. The eluent was concentrated under vacuum and stored at 4 °C. Gbt was purified from the eluent by semipreparative HPLC on a YMC 20 x 250 mm C18-AQ column, with a linear gradient of 20%–80% methanol (0.05% trifluoroacetic acid) over 40 min, yielding 20 mg from 1 L culture.

### Preparation of Isotopically Enriched Gbt

Isotopically enriched Gbt samples were isolated following the same protocols as nonlabeled Gbt. Amberlite® XAD-4 was freshly prepared for each isotopic study. ^15^N labeling of Gbt was accomplished using ^15^NH_4_Cl as the sole nitrogen source. Three amino acids (10 mM l-Arg, 10 mM l-Orn, and 10 mM l-Cit) were tested as possible substrates for conversion to Gra by addition to the ^15^NH_4_Cl MM9 medium (500 mL). For the preparation of ^13^C^15^N-Gra-enriched Gbt, 1 mM ^13^C^15^N-l-Arg was supplemented into MM9 as outlined above.

### Photolysis of Apo-Gbt

Photolysis of apo-Gbt was carried out in a 3 mL quartz cuvette with a 75 mm stir bar or in a quartz NMR tube. An Oriel Instruments Hg(Ar) (No. 6035) pen lamp was used as the UV source. Where mentioned, a bandpass filter (Edmund Optics 253.7 nm filter, 25.00 mm diameter, 40.00 nm full width at half maximum) was used to selectively irradiate at 253.7 nm. Samples were dissolved in an aqueous buffer (5 mM MOPS, 4-(2-hydroxyethyl)-1-piperazineethanesulfonic acid, or Na_2_HPO_4_) at pH 8.0. For NMR analysis, 99.9% purity D_2_O was used in place of Milli-Q H_2_O at pD 8.0.

### Photolysis of Fe-Gbt

Fe-Gbt was prepared in Na_2_HPO_4_ (25 mM, pH 8) in a 1:1 ratio of apo-Gbt and Fe(III) (FeCl_3_·6H_2_O, 2.58 ± 0.04 mM in 25 mM HCl) and was allowed to equilibrate for 20 h. The Fe(III) stock was standardized spectrophotometrically with 1,10 phenanthroline (510 nm, ε 1.1 × 10^4^ M^–1^ cm^–1^) and hydroxylamine as a reducing agent. Photolysis of Fe-Gbt was carried out in a quartz cuvette with a 75 mm stir bar. An Oriel Instrument Hg(Ar) (No. 6035) pen lamp was used as the UV source equipped with a bandpass filter (253.7 nm, Edmund Optics).

## Supporting Information Available

^1^H, ^13^C, HSQC, ^1^H–^13^C HMBC, ^1^H–^15^N HMBC, ^1^H–^1^H COSY, ^13^C–^13^C COSY, ^13^C INADEQUATE, and TOCSY NMR spectra, chemical shift tables, UPLC-MS spectra for isotopically labeled apo-Gbt and Gbt photoproduct, multiple sequence alignment of GrbD and GrbE, and biosynthesis of l-Arg (PDF)
