# Supplementary material for: C-Diazeniumdiolate Graminine in the Siderophore Gramibactin Is Photoreactive and Originates from Arginine
Source: ACS Chem Biol. 2022 Nov 10;17(11):3140–7. doi: 10.1021/acschembio.2c00593 (PMC9679993; doi:10.1021/acschembio.2c00593)
Supplement: Supplementary file 1 — cb2c00593_si_001.pdf [file cb2c00593_si_001.pdf]

Supporting Information

The *C*-diazeniumdiolate graminine in the siderophore gramibactin is photoreactive and originates from arginine

Christina Makris, † Jeffrey R. Carmichael, † Hongjun Zhou and Alison Butler*

Department of Chemistry & Biochemistry, University of California, Santa Barbara, CA 93106-9510 USA

* To whom correspondence may be addressed. butler@chem.ucsb.edu

† Equal co-authors

a)

```
grbD   369   ---AFAHRISARIDAECPGIDRETFVEPHEMCSTTHVHNDHRLVTIEEGDMLFWGNVGMQ  425
                 F      ++DA    +     VEP    S TH H+   L  I  G M F   +G
sznF   377   HDPVFEGLKQGKVDAPVAHL-----VEPRGELSNTHCHDGDELCHIVSGTMRFESGLGSS  431

grbD   426   HAMHKGDMVLIPDGRLHGSTVVSNECTY  453
               +  G+ V+I   RLHG+ + S+EC Y
sznF   432   LTLQAGEGVVIKRNRLHGANIESDECVY  459
```

b)

```
Mhr24    ----------------------------MTGPTTPGHEGAEDAVELFTEWLR--SDGFTC
DcsA     MNCYPAGHLLGKGHVQLNESGENAELISQEQIGDDLNGWHRDAFEDIASRLT--DPGFPC
GrbE     -MQAGNSAVGEADRFEVSDWKSRIIAGKATGAAVSAPAWLDASYATLREQVL--DPAYPC
AlpD     --------------------------MSRSGSGDEPFGWVPEAHSVFTERILAEEPPYPC

Mhr24    LGARAALHRGEL-VTGVYPALASAECLPELASDLGRFITGPLSTDGRFHSF--AAIFTEC
DcsA     VFSRNAFRKKLVKFVFVEGS--GKEDIRHLGAGLKDYVELSRDWDGALDTAYPLVVVFSA
GrbE     FFGTMAERRGEMFYSFVNGR-----DLRDLPATMQTFAELAVRPEYRRNN---IAVFFEP
AlpD     YFGTQGQQRGNNSFSAVDTRYPDTHGPAALARSLRAYRQ--RAWQGPKRQT--LIVFVGP

Mhr24    DPPDPDE-ETFEQLLWRQLRALHEADRTDNVWAHDVSDDPRSPDFGFSFGGHPFFVIGLH
DcsA     DAVTADSVEQYHAFGWWVLQELHAIDPTP--WPEGVDKGPQSEAWSMCFHGMPLFINMSS
GrbE     D-PEPLSHDAYRTLFWGILQRLHDIDPDP---AADQQPDPMDEAWEFSYAGVQMFVVCAC
AlpD     AVPGAELADDHRRF-WTLLDELRAYDTEP--WPADVPADPSDPRWQWCFDGEPWFIFAAS

Mhr24    PG-ASRVSRRFARP-ALVFNSHRQFAELKRAGVYQGMQTKIRAKERELQGD---VNPMLA
DcsA     PAHQVRRSRNLGRHFALVINPRERFDVFAGDTP-SGRKVRSNIRGRIARYDGTPHAQQLG
GrbE     PSFRARHSRNLGPGMVLLFQPRSVFVDTITNKV-IGREARNQVRKRLETWDDIPAHPDLG
AlpD     PAYKDRRSRDLGPCLTLVFQVRRVFEGIGGSTV-AGKAAKRRVREGLARYDRIGPHPTLG

Mhr24    EHGVIS--EARQY---SGRIVPPDWECPF----------------SRPG-----------
DcsA     SYGT-GALEWMQYGL-VEENRERADVCPF----------------TFRGA----------
GrbE     FYGDPGNLEWKQYFL-DDANAPIEERCPFLKRRAQAAAHSAQARRHVPAHGARNEHDKAF
AlpD     DGDTSTDFKWRQYTLPDDDSVAAPDACPV---RHHAAAPVLPERNAVDGSALPQPHERQR

Mhr24    -----------
DcsA     -----------
GrbE     AHWDDARGADN
AlpD     P----------
```

Figure S1. GrbD and GrbE sequence alignments**.** a) Sequence alignment of GrbD in Gbt and the cupin domain of SznF in the BGC of streptozocin.[1] Conserved residues are highlighted in blue. Residues in the active site of SznF that are not present in GrbD are highlighted in red. GrbD does not share sequence homology to the heme-dioxygenase-like (HDO) domain of SznF. There is a conserved switch to R and V in these positions in GrbD homologs in other reported and predicted diazeniumdiolate siderophores. Sequences aligned using NCBI BLAST. b) Sequence alignment of GrbE with other Fe-heme dependent Arg hydroxylases, Mhr24[2], DcsA[3], and AlpD[4]. The structures of these enzymes are not known, however they share a conserved cysteine, consistent with Cytochrome P450 heme-dependent enzymes. Sequences aligned with MUSCLE 3.8.

a) *P. graminis* culture aliquot: no irradiation

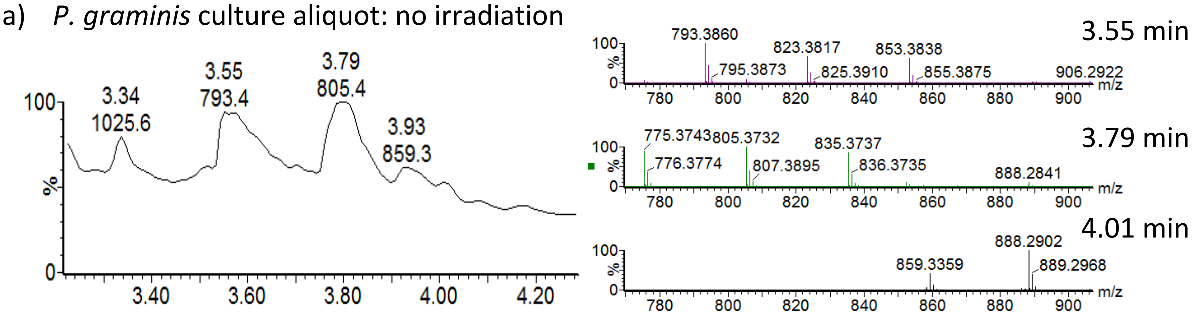

b) *P. graminis* culture aliquot: irradiation with sunlight

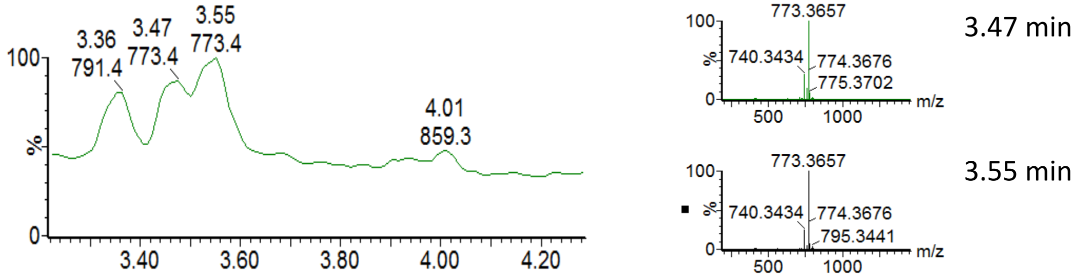

c) *P. graminis* culture aliquot: irradiation with Hg(Ar) pen lamp

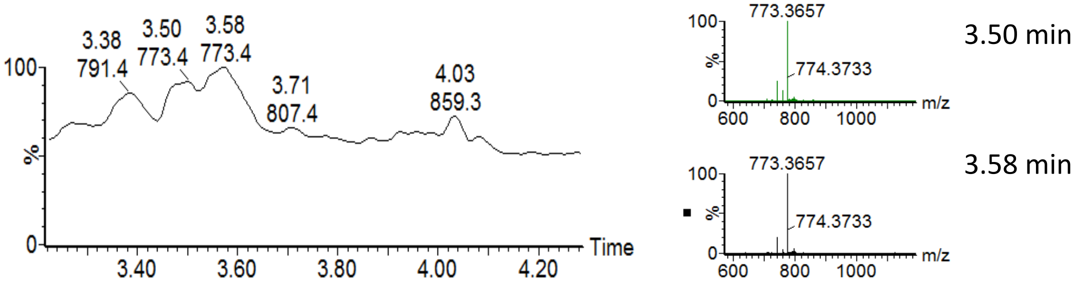

Figure S2. UPLC-MS total ion chromatograms of *P. graminis* DSM 17151 crude supernatant aliquots under dark and UV-exposed conditions. a) Control sample from *P. graminis* culture, with no UV light exposure showing presence of apo-Gbt (*m/z* 835 [*M*+H]$^+$) at 3.79 min, linear apo-Gbt (853 [*M*+H]$^+$) at 3.55 min and Fe(III)-Gbt (*m/z* 888 [*M*-2H+Fe]$^+$ at 4.0 min; b) *P. graminis* culture aliquot irradiated with sunlight for 6 hours showing formation of E/Z oxime isomer photoproducts (*m/z* 773 [*M*+H]$^+$) at 3.47 min and 3.55 min; c) *P. graminis* culture aliquot irradiated with Hg(Ar) pen lamp (254 nm) for 1 hour showing E/Z oxime isomer photoproducts (*m/z* 773 [*M*+H]$^+$) at 3.50 and 3.58 min.

a)

$$4NO + O_2 + 2H_2O \longrightarrow 4\ NO_2^- + 4\ H^+$$

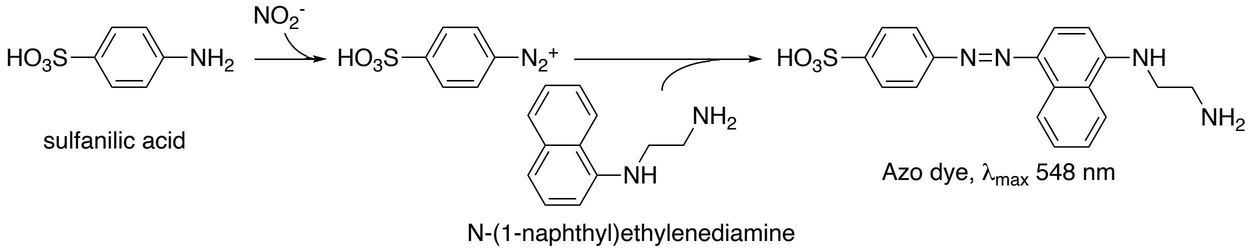

b)

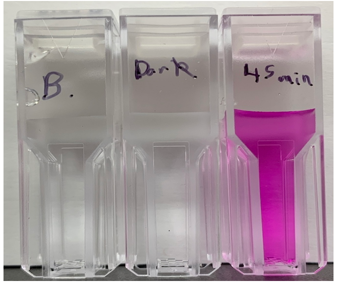

Figure S3. a) Reaction scheme for NO oxidation and the Griess test for nitrite detection.[5-7]
b) UV-photolysis of apo-Gbt: left, blank without Gbt; middle, apo-Gbt kept in the dark; photolyzed apo-Gbt showing a positive colorimetric Griess response, indicating detection of NO_2^- which is produced from labilization of NO and subsequent oxidation of NO to NO_2^-.

For the Griess assay, equal volumes of 1% (w/v) sulfanilic acid (5% (w/v) H_3PO_4) and 0.1% (w/v) N-(1-naphthyl)-ethylenediamine (in H_2O) were combined. A 50 μL aliquot was added to a 300 μL nitrite-containing sample and dilute to 1.5 mL with Milli-Q H_2O. The solution was allowed to sit for 60 minutes and then the absorbance was measured at 548 nm.[5-7] A blank was prepared with 300 μL Milli-Q H_2O.

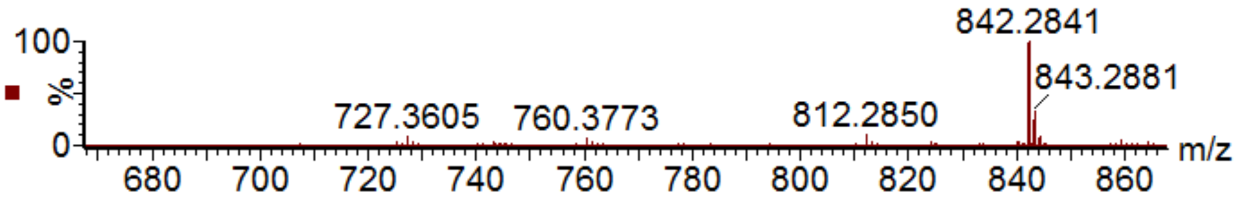

Figure S4. UPLC-MS after 20 min UV irradiation of Fe(III)-Gbt (pH 8.0, 25 mM Na_2HPO_4; Hg(Ar) pen lamp with 253.7 nm bandpass filter) with most prominent photoproduct at *m/z* 842, consistent with loss of CO_2 and 2 H^+'s from β-OH-Asp of Fe(III)-Gbt.

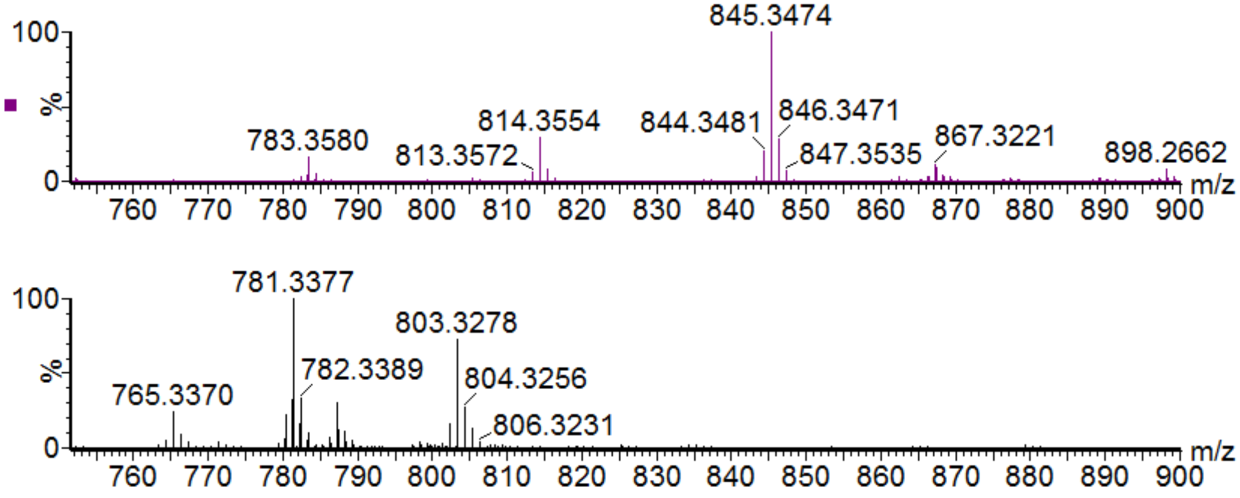

Figure S5. Mass spectra of (top) fully ^{15}N-enriched Gbt *m/z* 845.3 [*M*+H]$^+$ (M+10) with ionization-induced mass losses of 31 (-^{15}NO) and (bottom) photolyzed ^{15}N-apo-Gbt showing the photoproduct (*m/z* 781.3 [*M*+H]$^+$) with mass loss of 64 (2 ^{15}NO + 2 H$^+$).

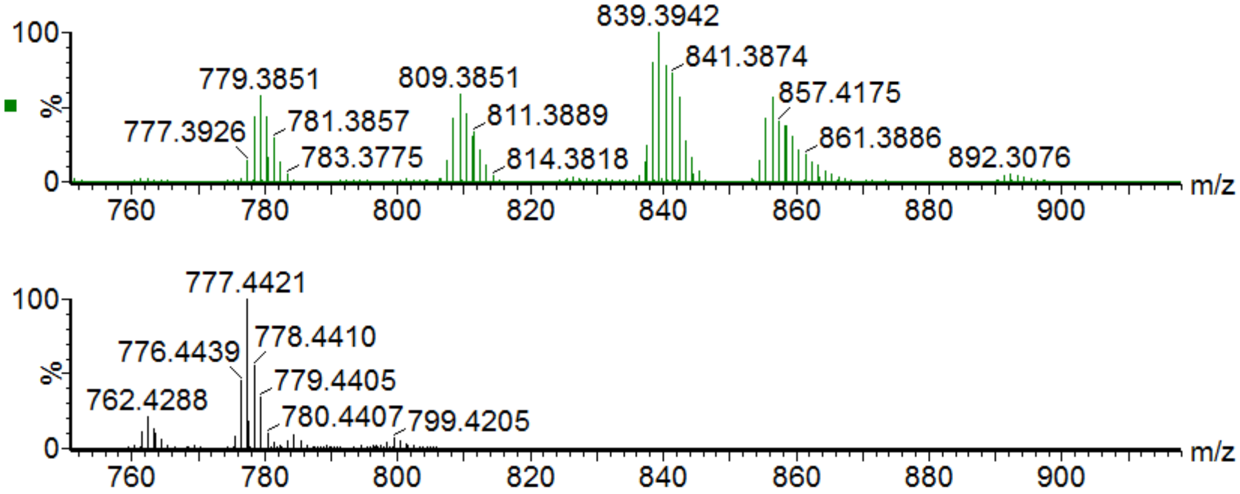

Figure S6. Mass spectrum of apo-Gbt (top) isolated from *P. graminis* DSM 17151 grown in $^{15}NH_4Cl$ and ^{14}N-Arg supplemented medium shows *m/z* 839.3 [*M*+H]$^+$ (M+4) with mass losses of 30 (-^{14}NO). The M+4 isotopically labeled Gbt photoproduct (*m/z* 777 [*M*+H]$^+$, bottom) shows a mass loss of 62 (2 ^{14}NO + 2 H$^+$).

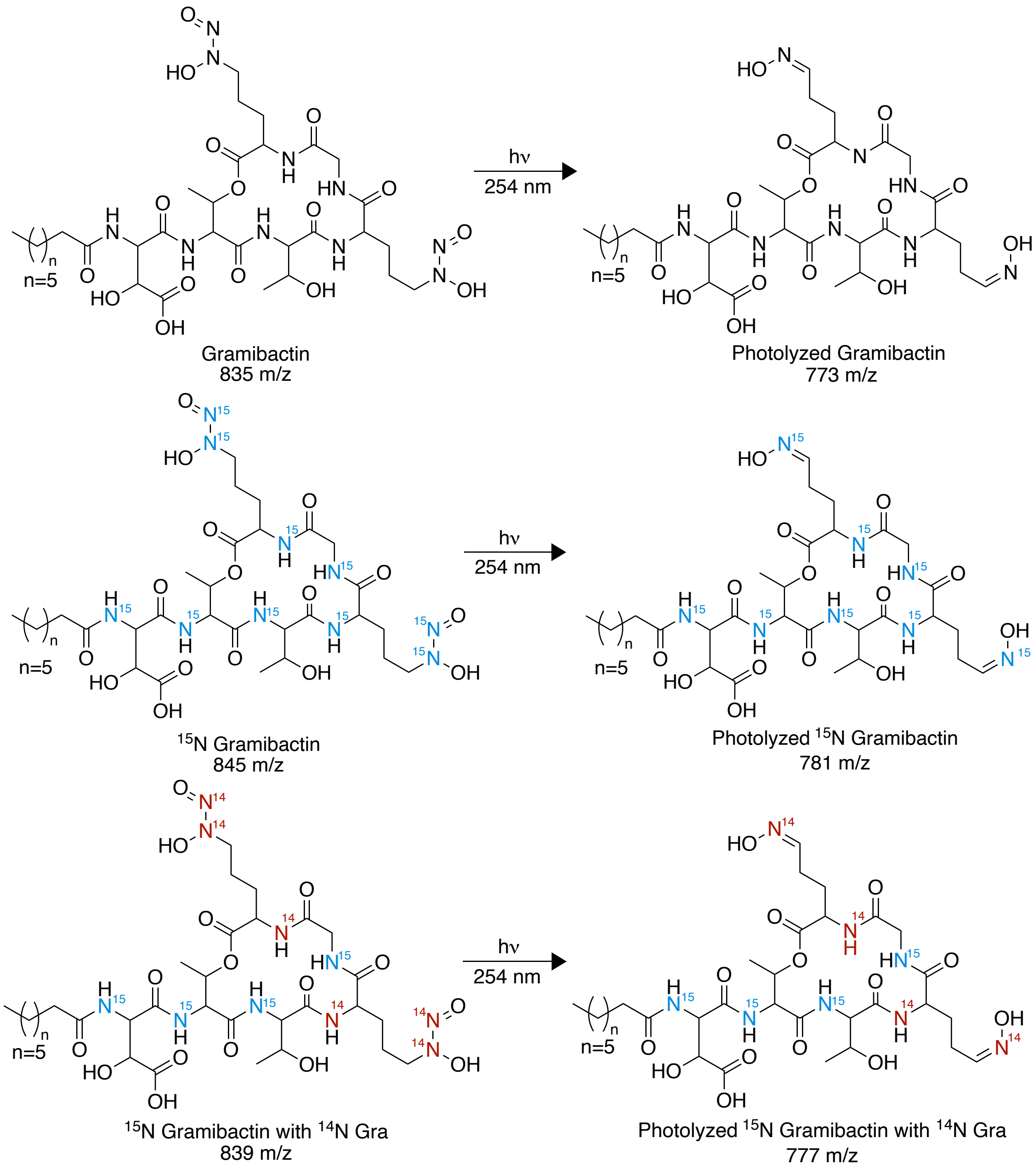

Figure S7. Photolysis of Gbt and isotopically enriched-Gbt. ^{15}N-Gra-enriched-Gbt observe a mass loss of 64 in photoproduct, while ^{14}N-Gra-enriched-Gbt show a mass loss of 62.

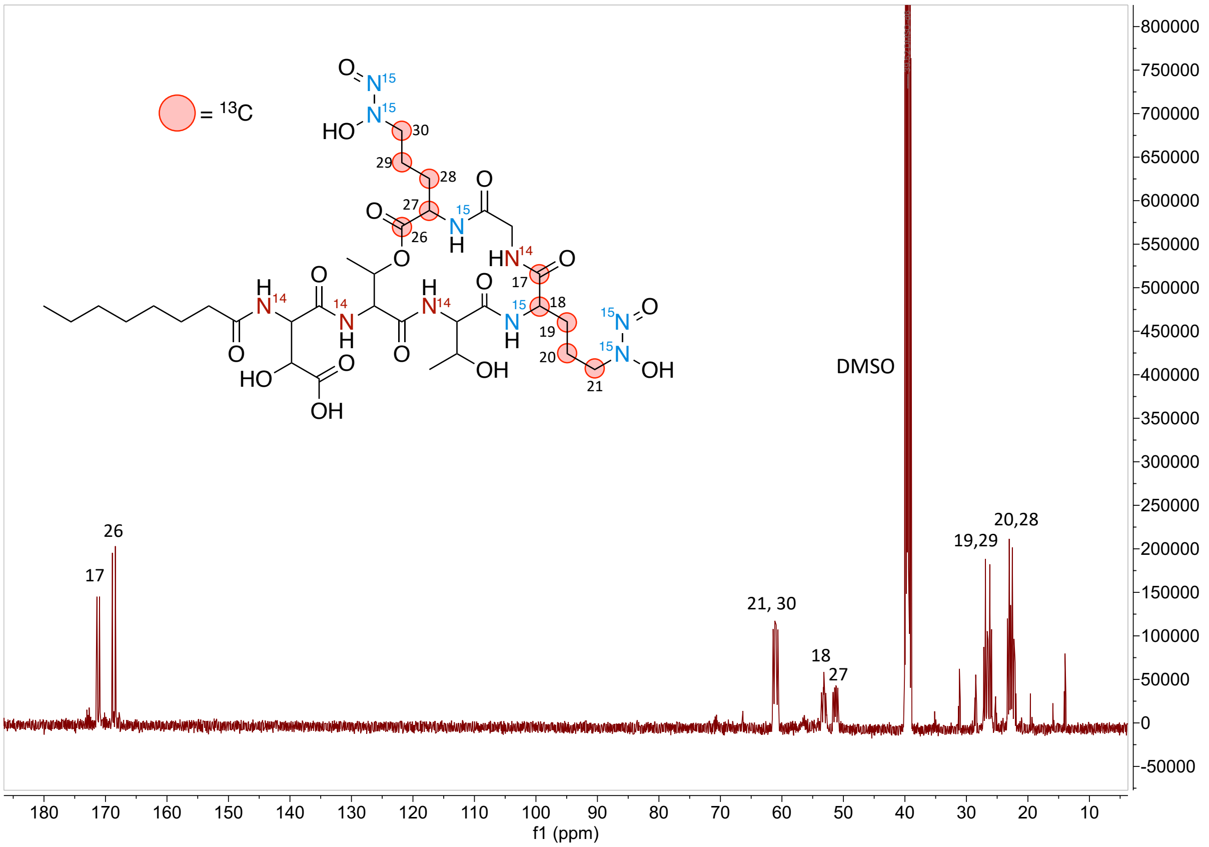

Figure S8. ^{13}C NMR characterization of Gbt isolated from *P. graminis* DSM 17151 supplemented with ^{13}C^{15}N Arg. The ^{13}C NMR spectrum shows full incorporation of labeled Arg in Gra residues. Spectrum acquired on Bruker 500 in DMSO-d_6.

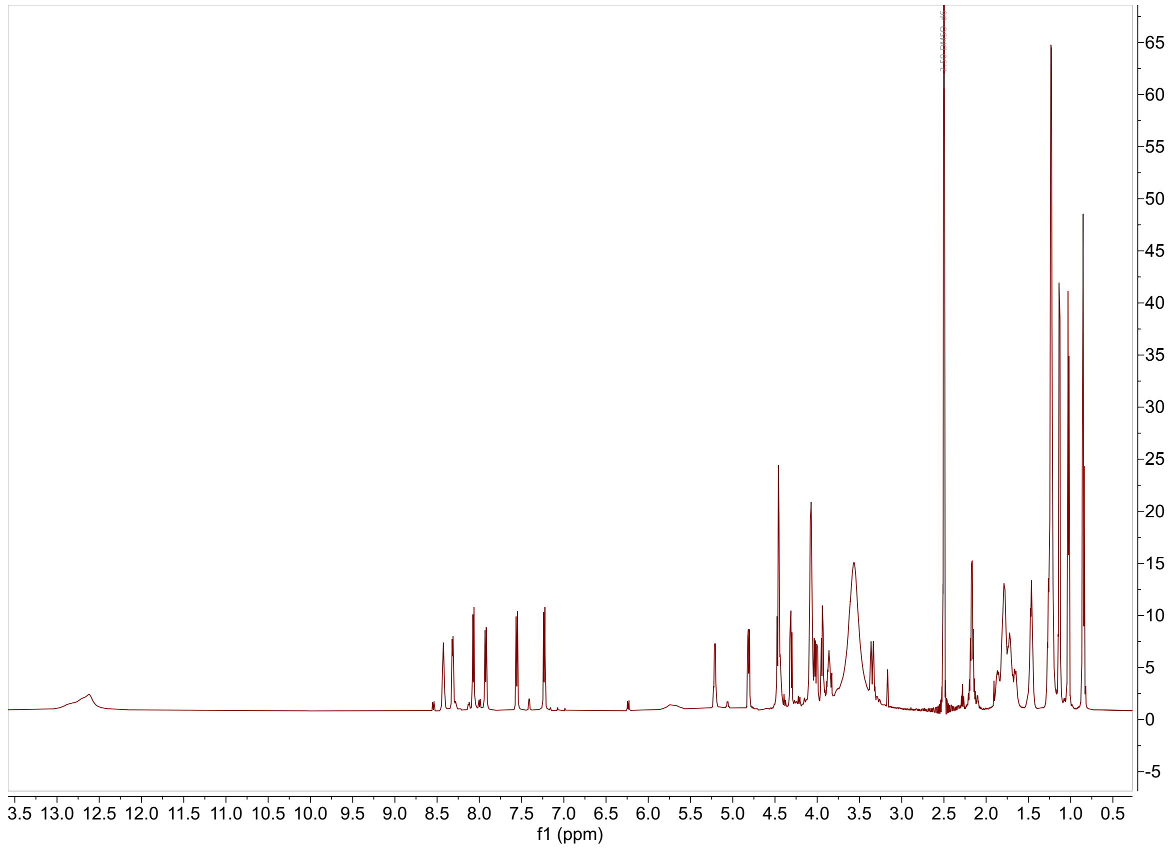

Figure S9. ^{1}H NMR of ^{13}C^{15}N-Gra-enriched-Gbt with ^{13}C (40 ppm) and ^{15}N-decoupling (110 ppm). Spectrum acquired on Varian 600 in DMSO-d_6.

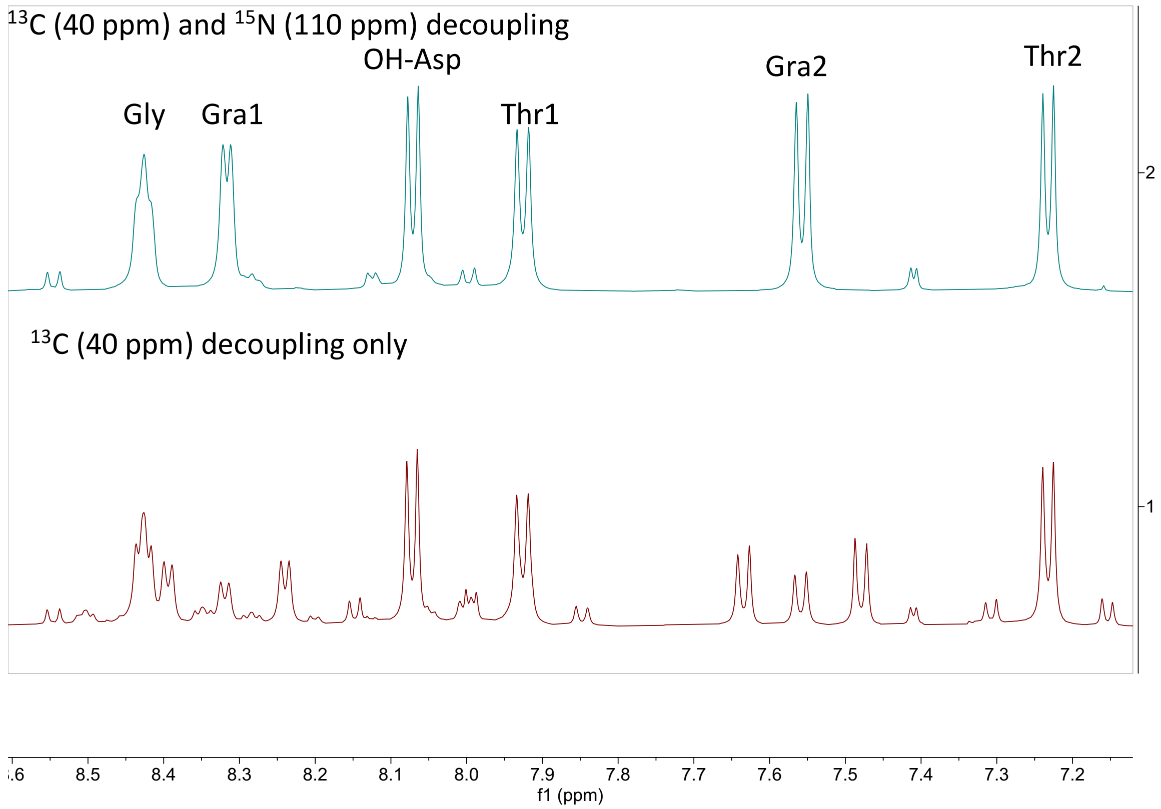

Figure S10. ^{1}H NMR spectrum (bottom) of the Gbt amide region shows some incorporation of ^{15}N in amides in the peptide backbone of Gbt, which is derived from the supplemented $^{13}C^{15}N$ Arg. $^{15}NH_3$ may be released in the process of conversion of Arg to Gra or as a byproduct of arginase and the urea cycle. J coupling constants of ~ 90 Hz between each of the ^{15}N satellite peaks are observed in the absence of ^{15}N decoupling (bottom). ^{14}N-Gra residues are also evident, based on the presence of an amide proton signals with ^{15}N decoupling (top). Spectra were acquired on Varian 600 in DMSO-d_6.

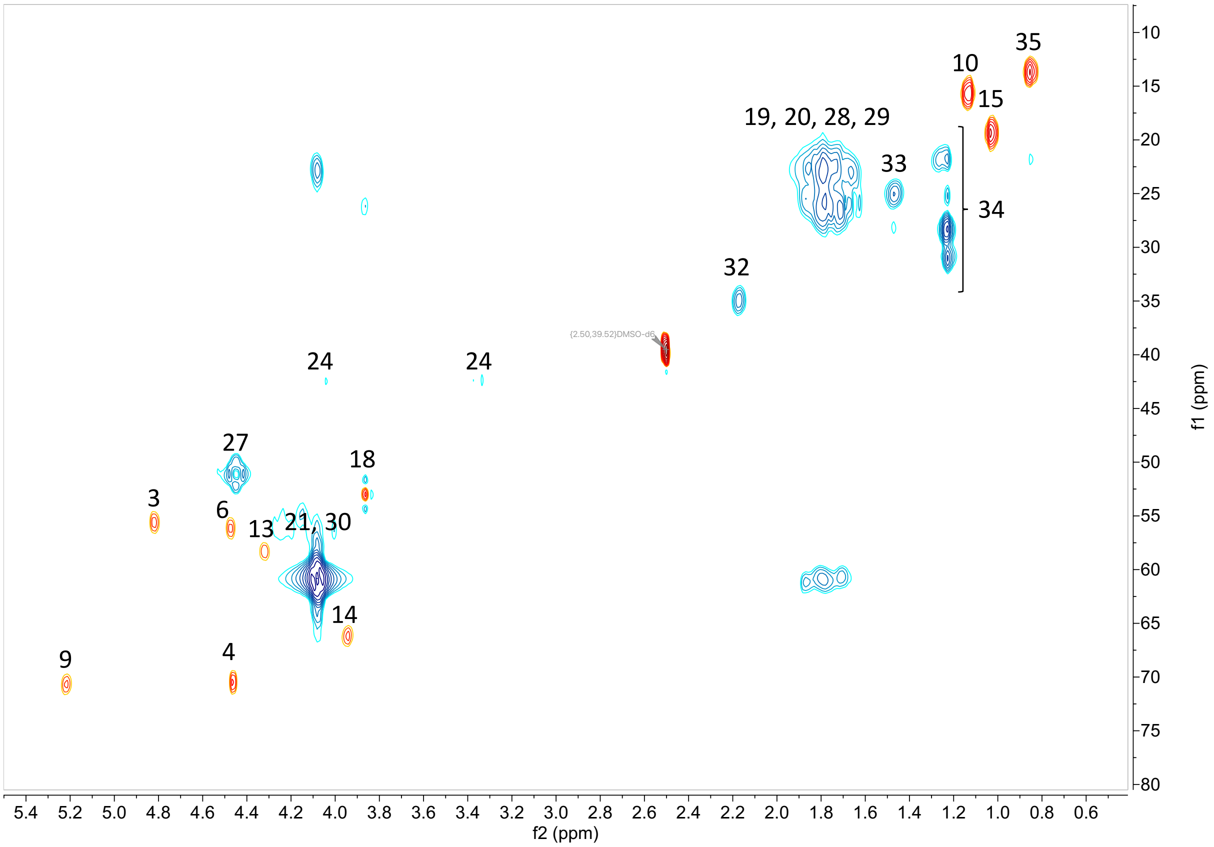

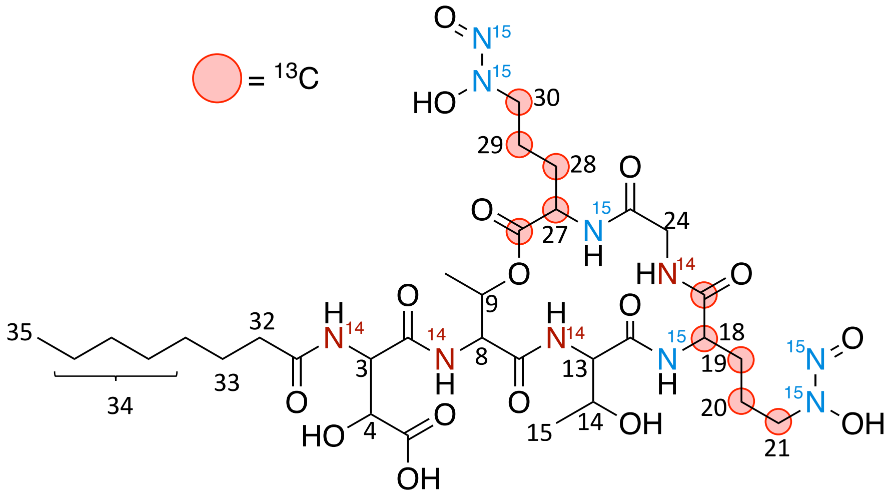

Figure S11. Multiplicity edited HSQC NMR experiment supports structural characterization of $^{13}C^{15}N$-enriched-Gra residues in Gbt (red: CH or CH_3, blue: CH_2). Strong but broad HSQC signals from $^{13}C^{15}N$-enriched-Gra residues are apparent in comparison to naturally ^{13}C abundant nuclei. Broadening of isotopically enriched signals is due to J-coupling between adjacent ^{13}C and ^{15}N spins. Spectrum acquired on Bruker 500 in DMSO-d_6.

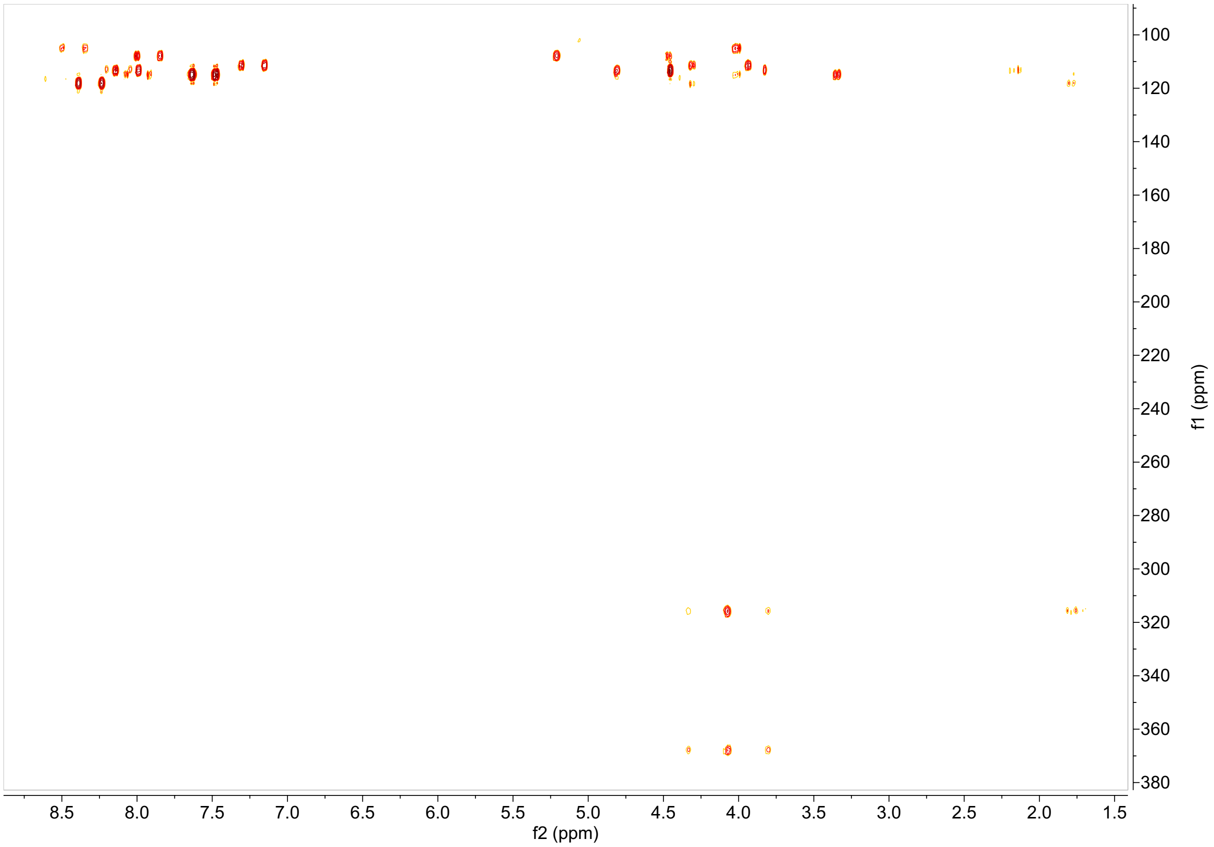

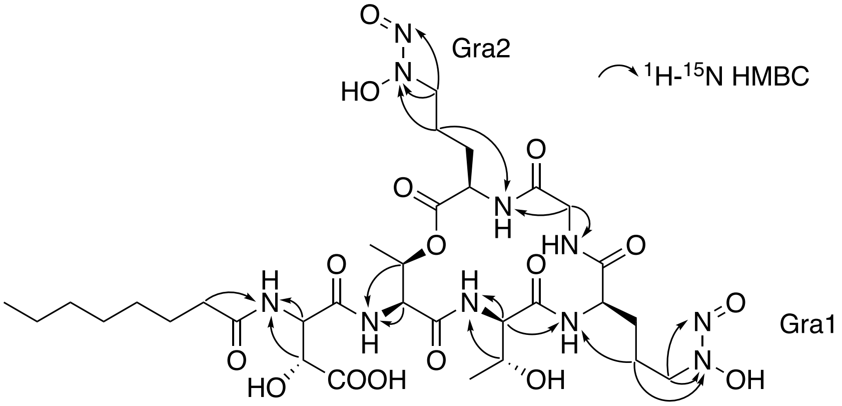

Figure S12. ^{1}H-^{15}N HMBC (with ^{13}C-decoupling during acquisition, 40 ppm) spectrum of ^{13}C^{15}N-enriched-Gra in Gbt. Spectrum acquired on Varian 600 in DMSO-d_6.

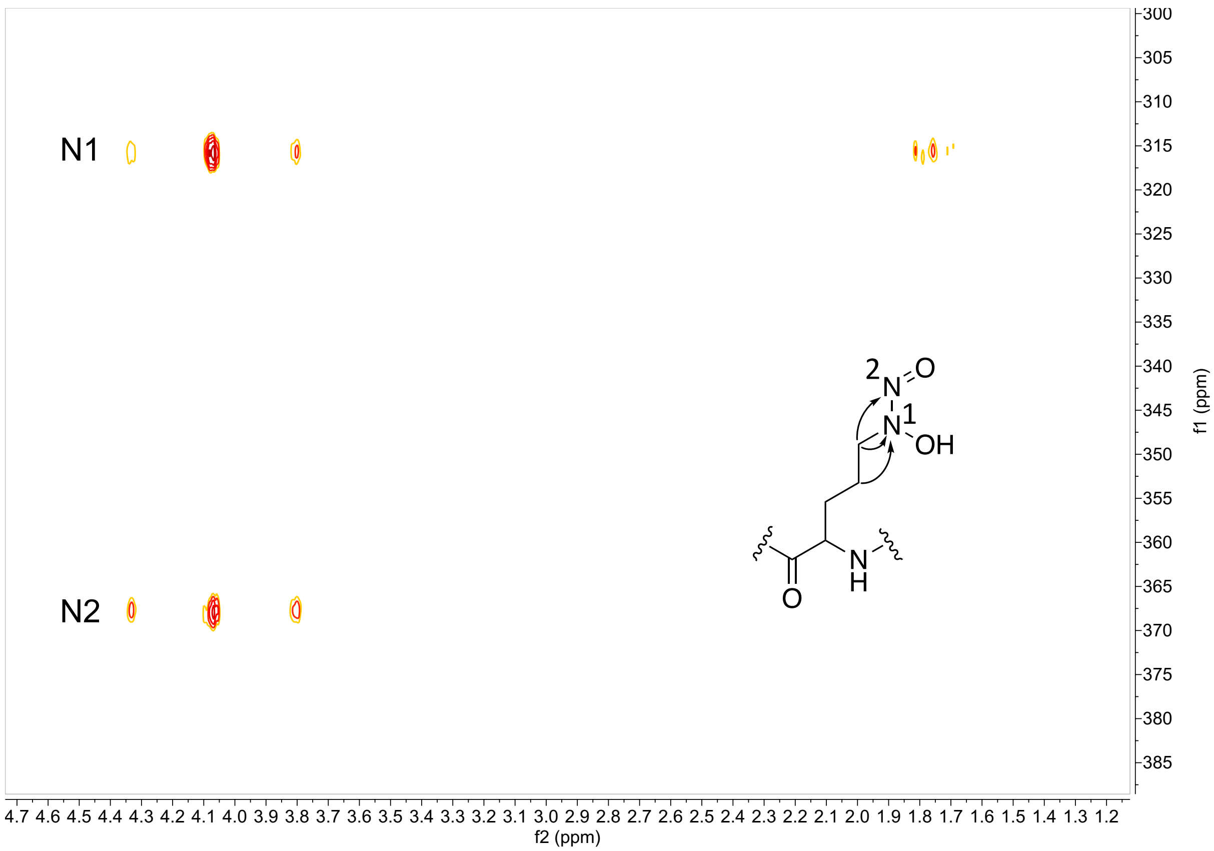

Figure S13. ^{1}H-^{5}N HMBC (with ^{13}C-decoupling during acquisition) spectrum of ^{13}C^{15}N-enriched-Gra in Gbt shows the presence of a *C*-diazeniumdiolate in the two Gra residues. The Cγ protons of Gra1 and Gra2 only correlate to Nitrogen 2 while the Cδ protons correlate to both nitrogen spins. Spectrum acquired on Varian 600 in DMSO-d_6.

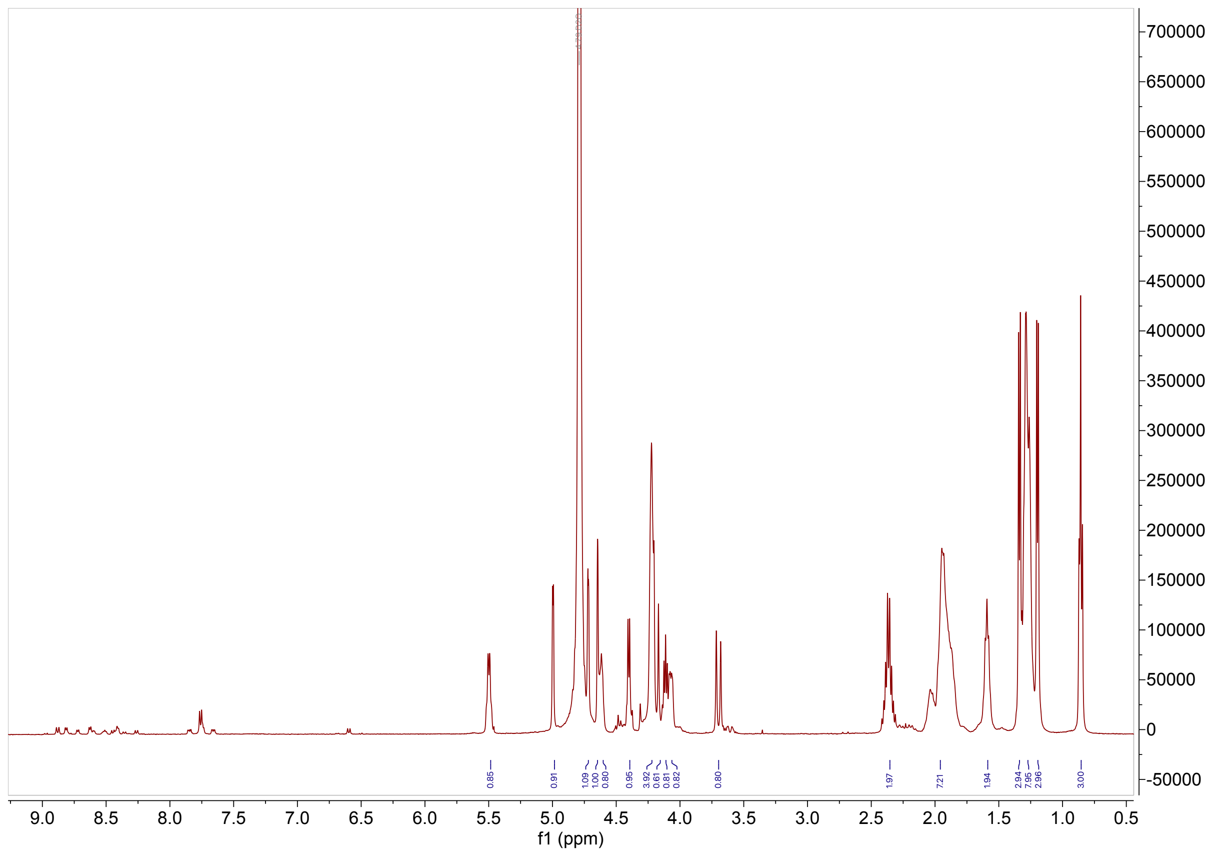

Figure S14. ^{1}H NMR spectrum of $^{13}C^{15}N$-enriched-Gra in Gbt. Spectrum acquired on Bruker 500 in D_2O.

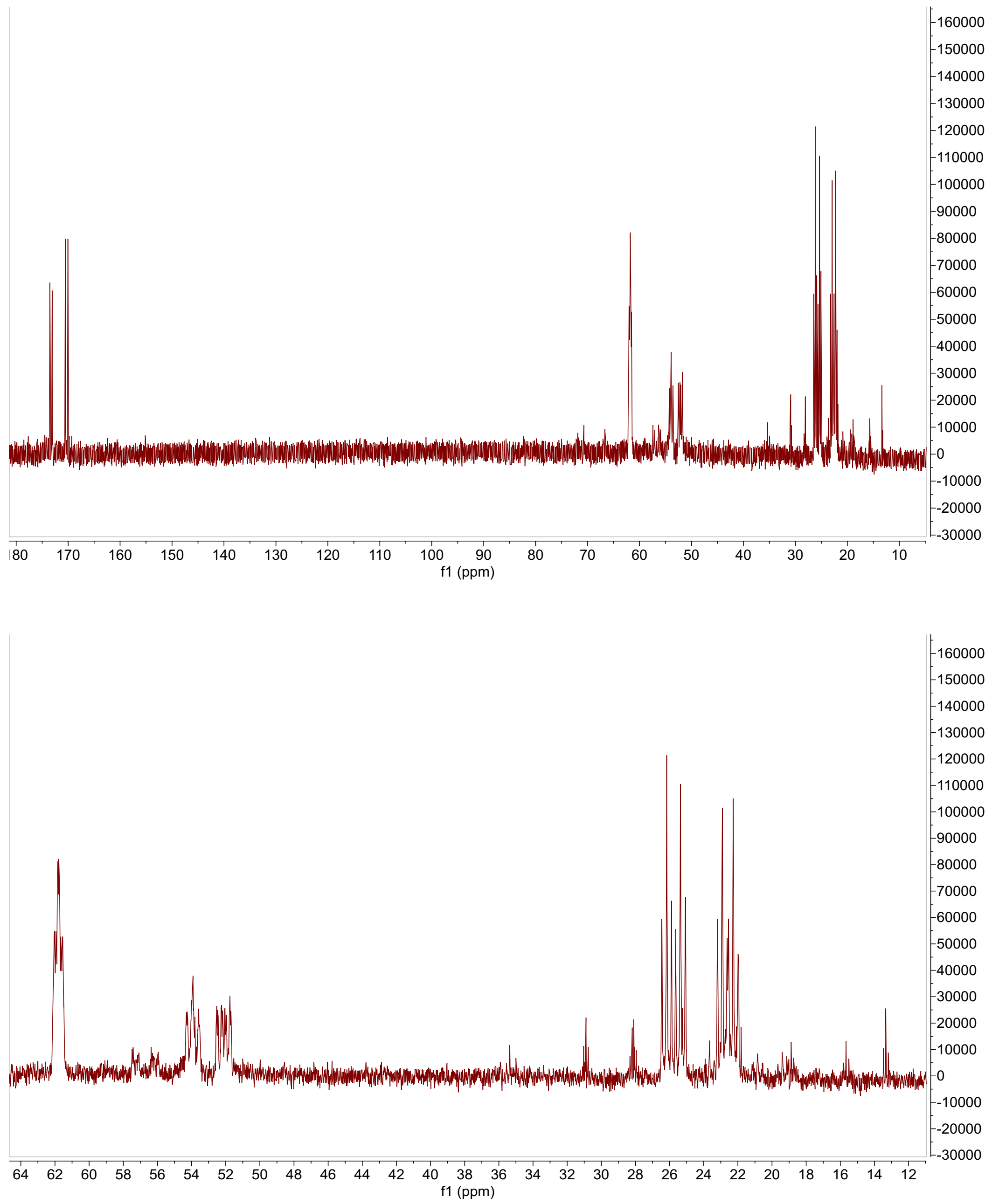

Figure S15. Top: ^{13}C NMR of $^{13}C^{15}N$-Gra-enriched-Gbt. Bottom: Expanded region from 10 to 64 ppm. Spectrum acquired on Bruker 500 in D_2O.

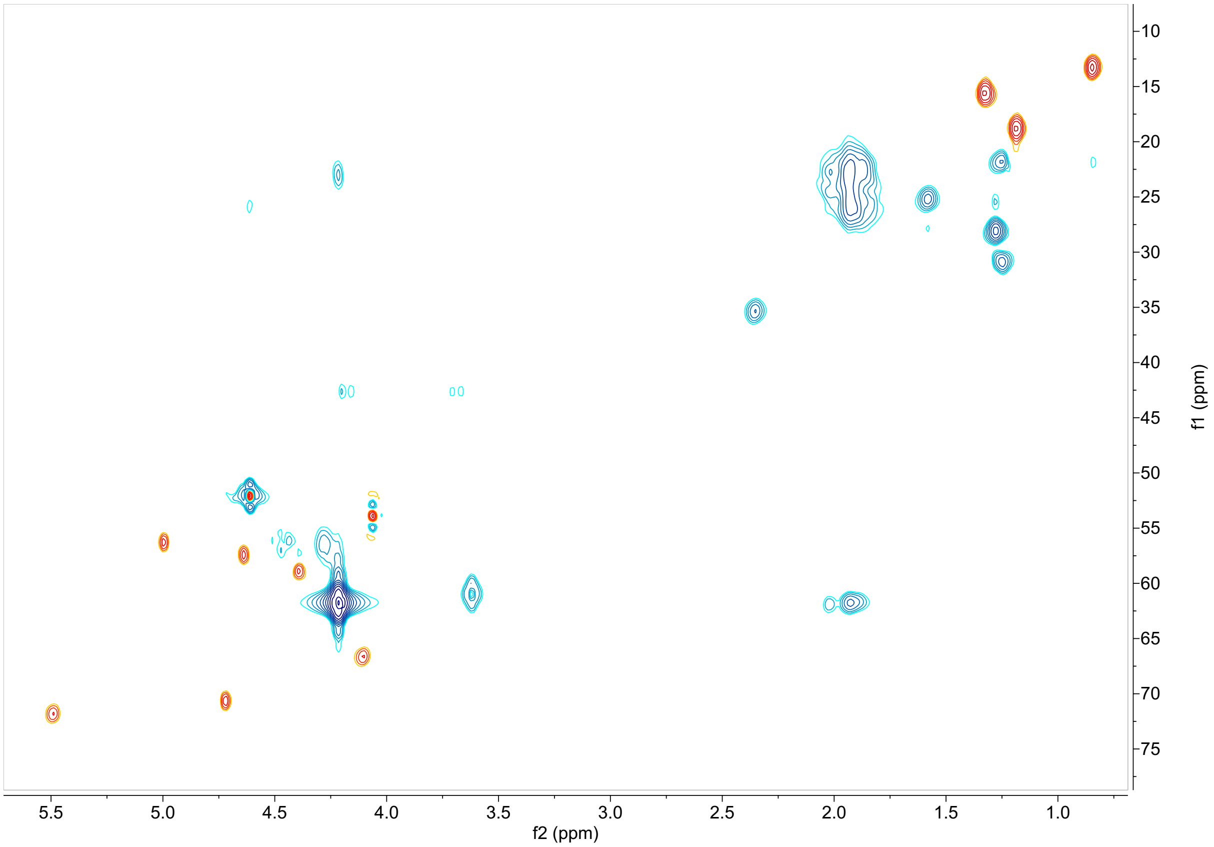

Figure S16. Multiplicty edited HSQC spectrum of $^{13}C^{15}N$-Gra-enriched-Gbt (red: CH or CH_3, blue: CH_2). Strong but broad HSQC signals from $^{13}C^{15}N$-enriched-Gra residues are apparent in comparison to naturally ^{13}C abundant nuclei in D-*allo* Thr, L-Thr, D-*threo*-OH-Asp, and Gly. Broadening of isotopically enriched signals is due to J-coupling between adjacent ^{13}C and ^{15}N spins. Spectrum acquired on Bruker 500 in D_2O.

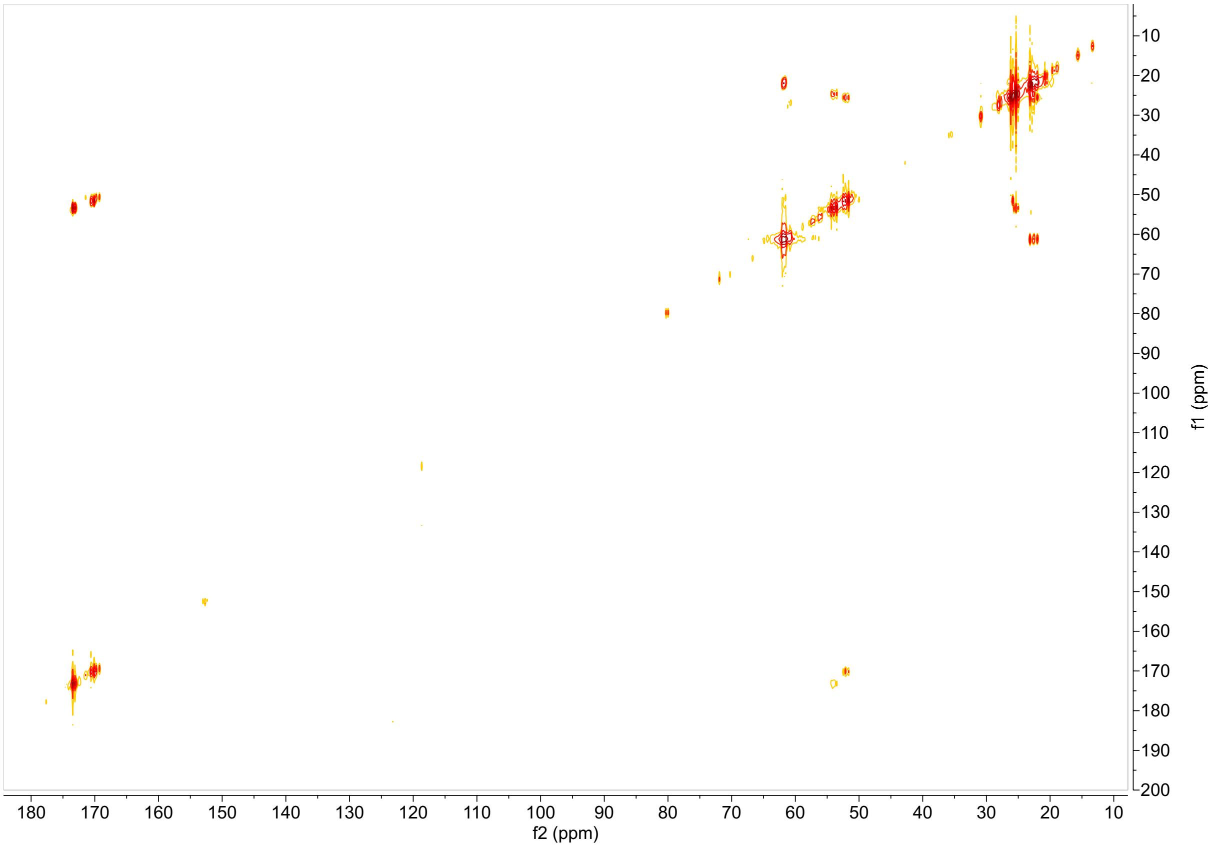

Figure S17. ^{13}C-^{13}C COSY NMR experiment on $^{13}C^{15}N$-enriched-Gra in Gbt shows direct correlations between all ^{13}C's in each Gra, which are all derived from $^{13}C^{15}N$-Arg. Resonances from natural abundant 13C are not expected to be observed in this insensitive experiment. Spectrum acquired on Bruker 500 in D_2O.

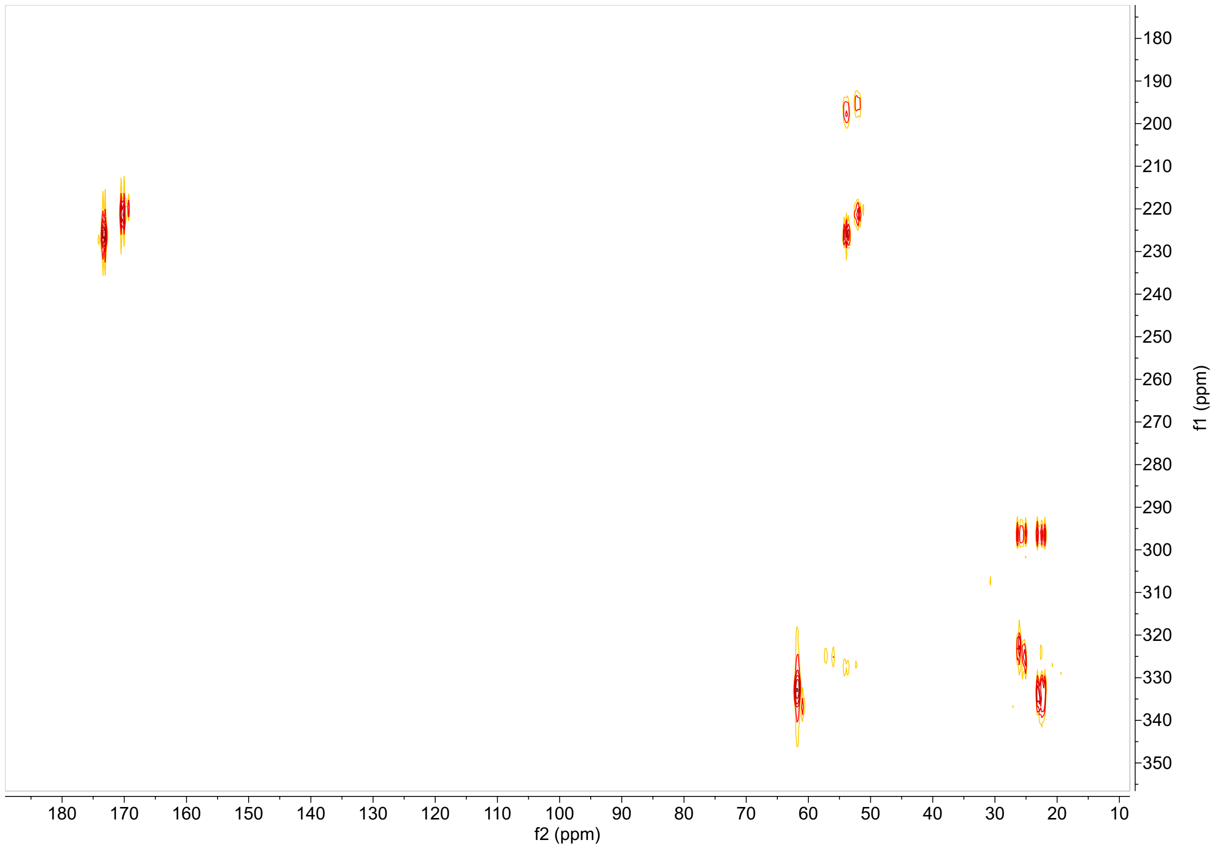

Figure S18. ^{13}C INADEQUATE (Incredible Natural Abundance DoublE QUAntum Transfer Experiment) NMR spectrum shows direct correlation of all ^{13}C-enriched-Gra carbons in Gbt isolated from $^{13}C^{15}N$-Arg supplemented *P. graminis* DSM 17151. Resonances from natural abundant ^{13}C are not expected to be observed in this insensitive experiment. Spectrum acquired on Bruker 500 in D_2O.

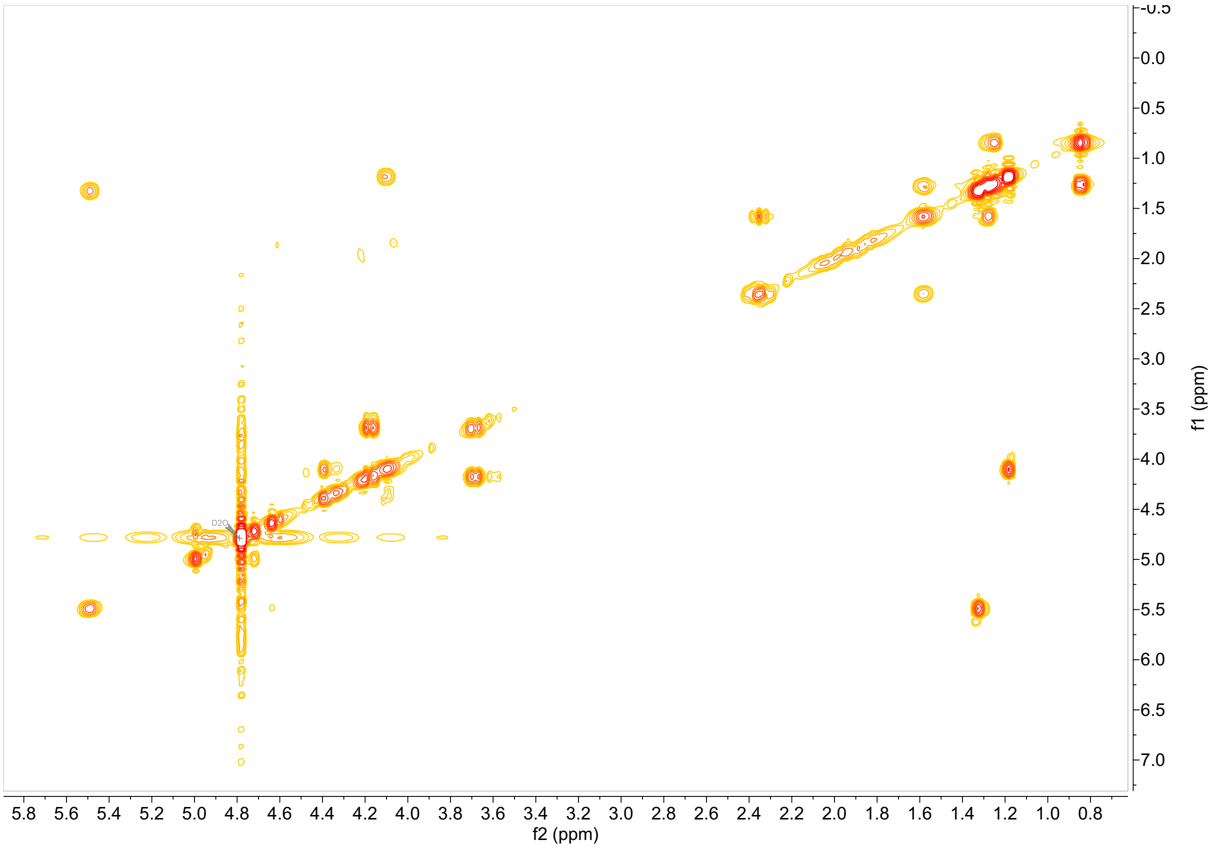

Figure S19. ^{1}H-^{1}H COSY NMR spectrum of $^{13}C^{15}N$-Gra-enriched-Gbt. Spectrum acquired on Bruker 500 in D_2O.

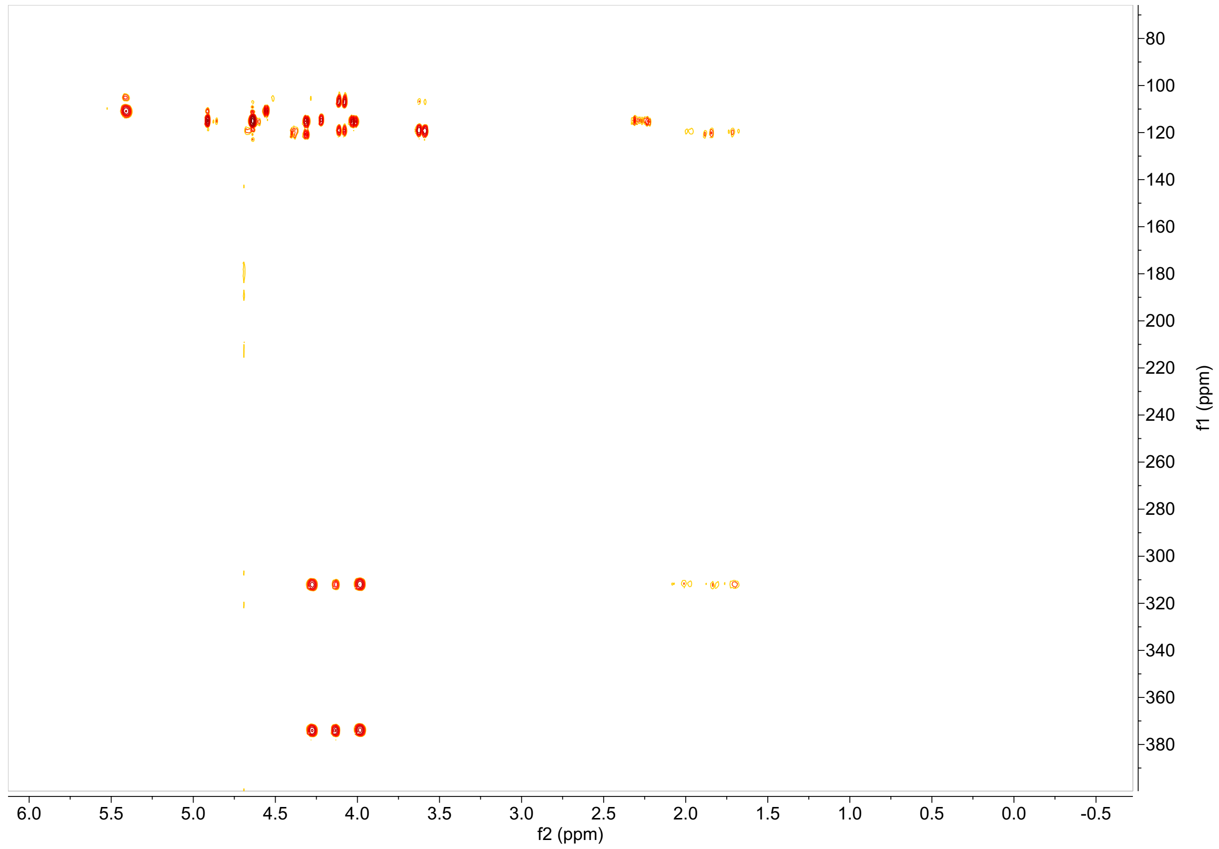

Figure S20. ^{1}H-^{15}N HMBC NMR spectrum of $^{13}C^{15}N$-Gra-enriched-Gbt. Spectrum acquired on Varian 600 in D_2O.

a. Biosynthesis of arginine

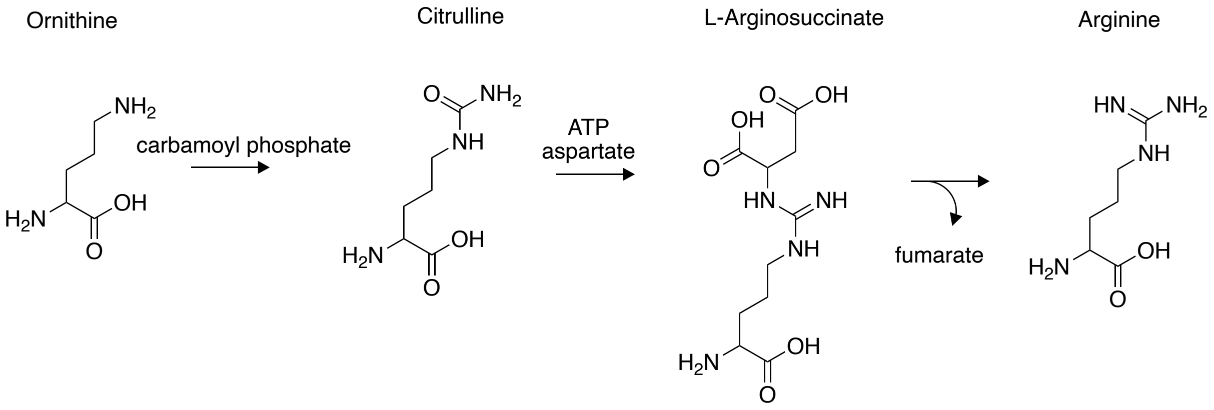

b. Urea cycle converts arginine back to ornithine

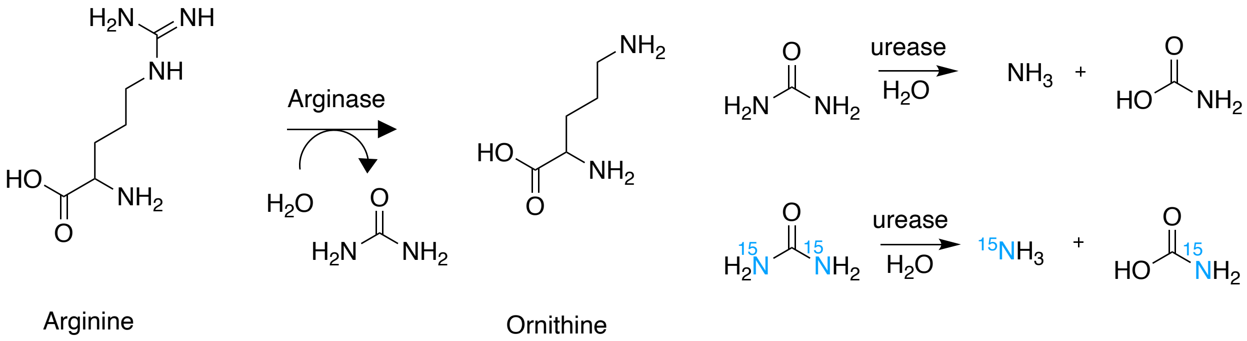

Figure S21. Biosynthesis of L-Arg. As part of the urea cycle, L-Arg is converted to L-Orn by an arginase. In the process, urea is formed which is further hydrolyzed to ammonia by a urease.[8-10] Cultures of *P. graminis* DSM 17151 supplemented with $^{13}C^{15}N$-Arg produced $^{15}NH_3$, either via arginase and the urea cycle or as a byproduct of the conversion from Arg to Gra; released $^{15}NH_3$ can be incorporated into the D-*threo*-β-OH-Asp, Gly, and L-Thr, and D-*allo*-Thr peptidyl backbone amides in Gbt. This ^{15}N incorporation in the peptide backbone yields the observed M+17 isotopic mass (*m/z* 852.3) for incorporation of one equivalent of $^{15}NH_3$. ^{1}H-^{15}N coupling (J ~ 90 Hz) is present in the amide region of the ^{1}H NMR.[11]

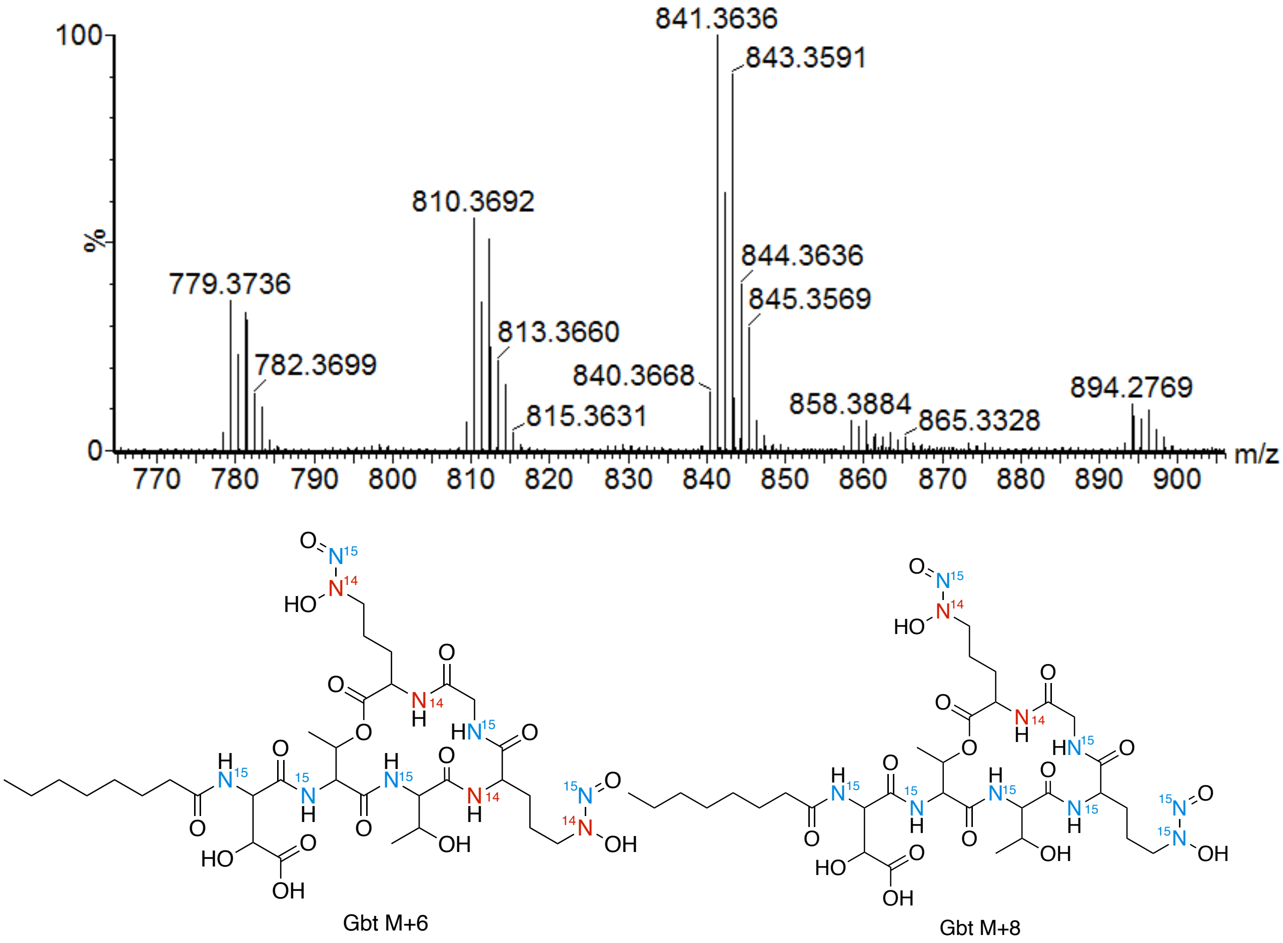

Figure S22. *P. graminis* DSM 17151 cultured with $^{15}NH_4Cl$ and ^{14}N-L-Orn shows two major isotopically labeled Gbt compounds at *m/z* 841.3 $[M{+}H]^+$ (M+6) and *m/z* 843.3 $[M{+}H]^+$ (M+8). L-Orn is on the microbial biosynthetic pathway to L-Arg and must obtain a nitrogen from carbamoyl phosphate, leading ultimately to one ^{15}N per Gra derived from L-Orn. It was deduced that the ^{15}N-enriched nitrogen in the Gra residue of the resulting Gbt must be the nitroso nitrogen since mass losses of 31 are observed.

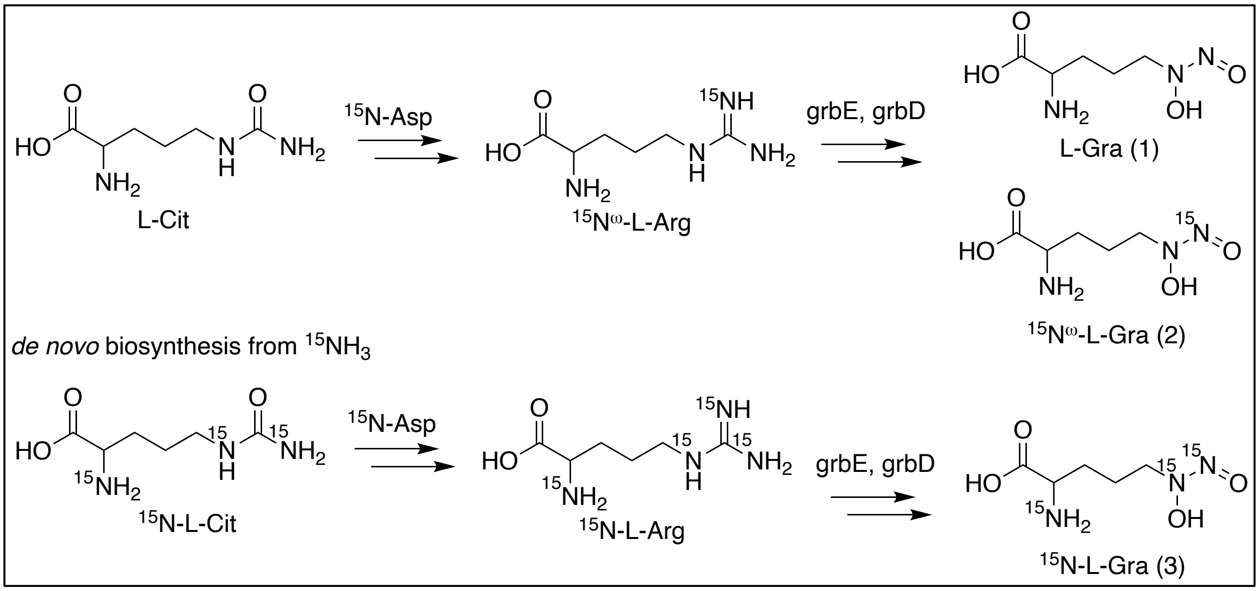

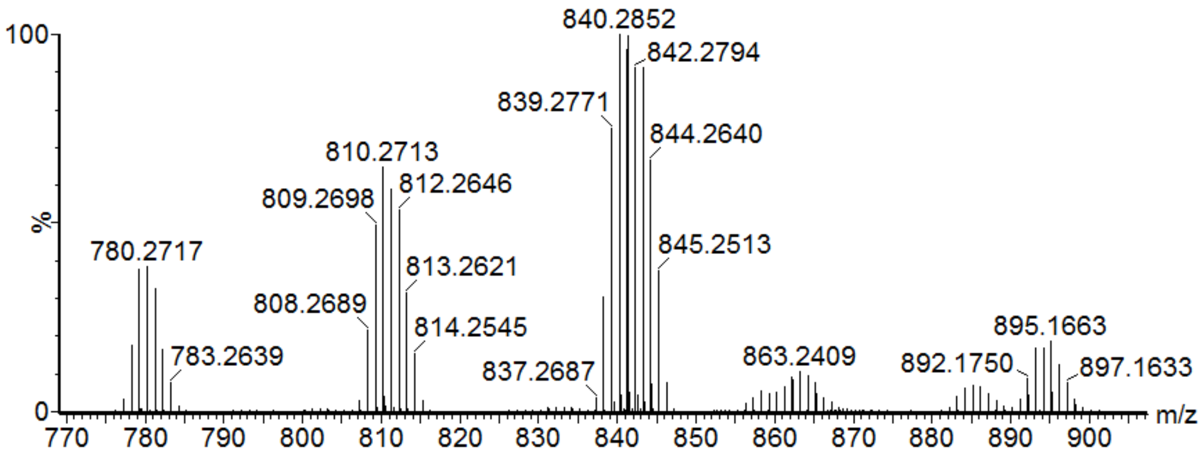

Gra1	Gra2	M+	*m/z* $[M+H]^+$
1	1	M+4	839
1	2	M+5	840
2	2	M+6	841
3	1	M+7	842
3	2	M+8	843
3	3	M+10	845

Figure S23. MS of Gbt isolated from *P. graminis* DSM 17151 cultured with $^{15}NH_4Cl$ and ^{14}N-L-Cit shows a range of isotopic masses. On the microbial biosynthetic pathway to L-Arg, L-Cit incorporates a nitrogen from L-Asp, in this case ^{15}N-L-Asp. In the rearrangement of Arg to Gra, one nitrogen must be lost. The two N^{ω} in the guanidinium group of L-Arg are equivalent, so in this case one will be ^{15}N labeled, thus leading to two possible Gra residues as shown above as 1 and 2. Gra might also be synthesized from the $^{15}NH_4$ also fed to the culture giving six possible Gbt isotopes, all of which are observed.

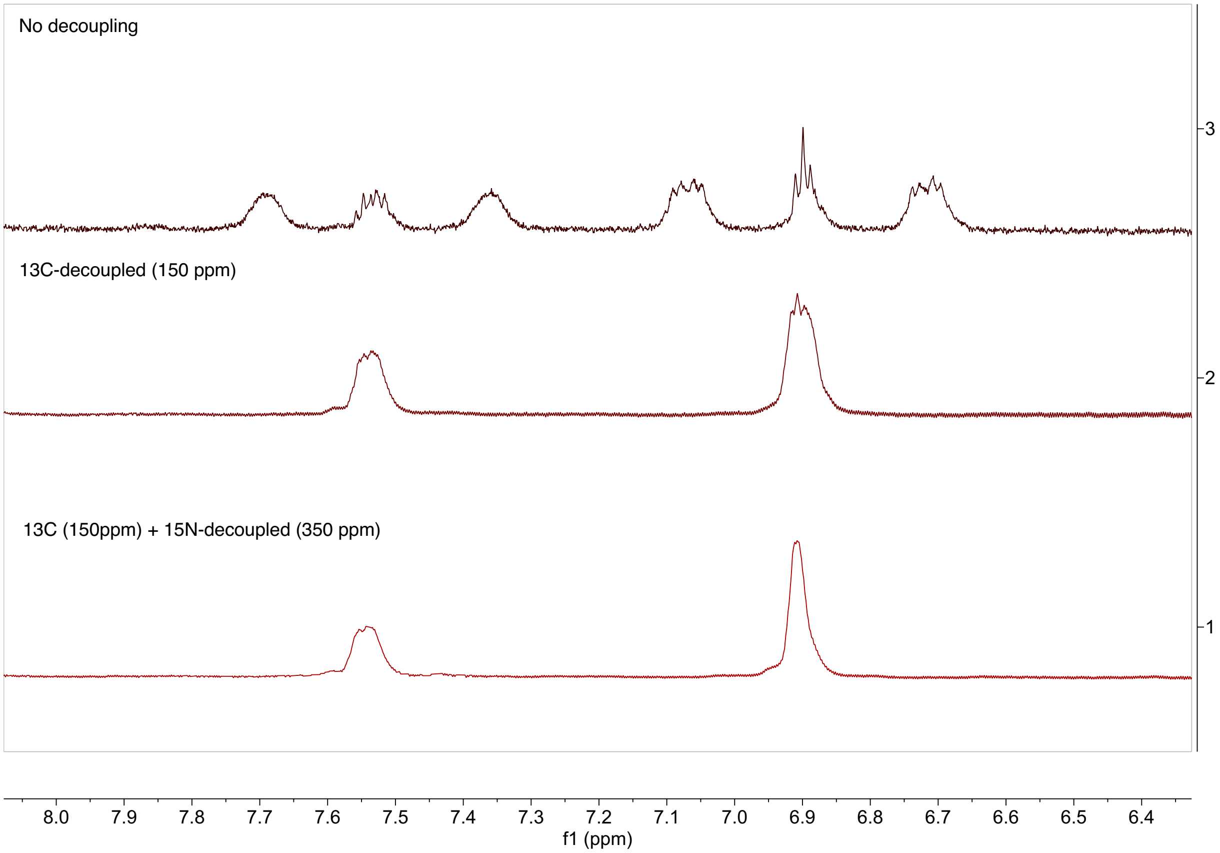

Figure S24. E/Z oxime ^{1}H resonances appear after photolysis of $^{13}C^{15}N$-Gra-enriched Gbt. Protons on the $^{13}C\delta$ of Gra are adjacent to ^{15}N and $^{13}C\gamma$. Decoupling of ^{13}C and ^{15}N shows a narrower ^{1}H resonance (bottom). E/Z oxime isomers exhibit a large ppm difference (0.6 ppm), with the E isomer being more deshielded than the Z isomer.[12, 13] Spectra collected on Varian 600 in D_2O.

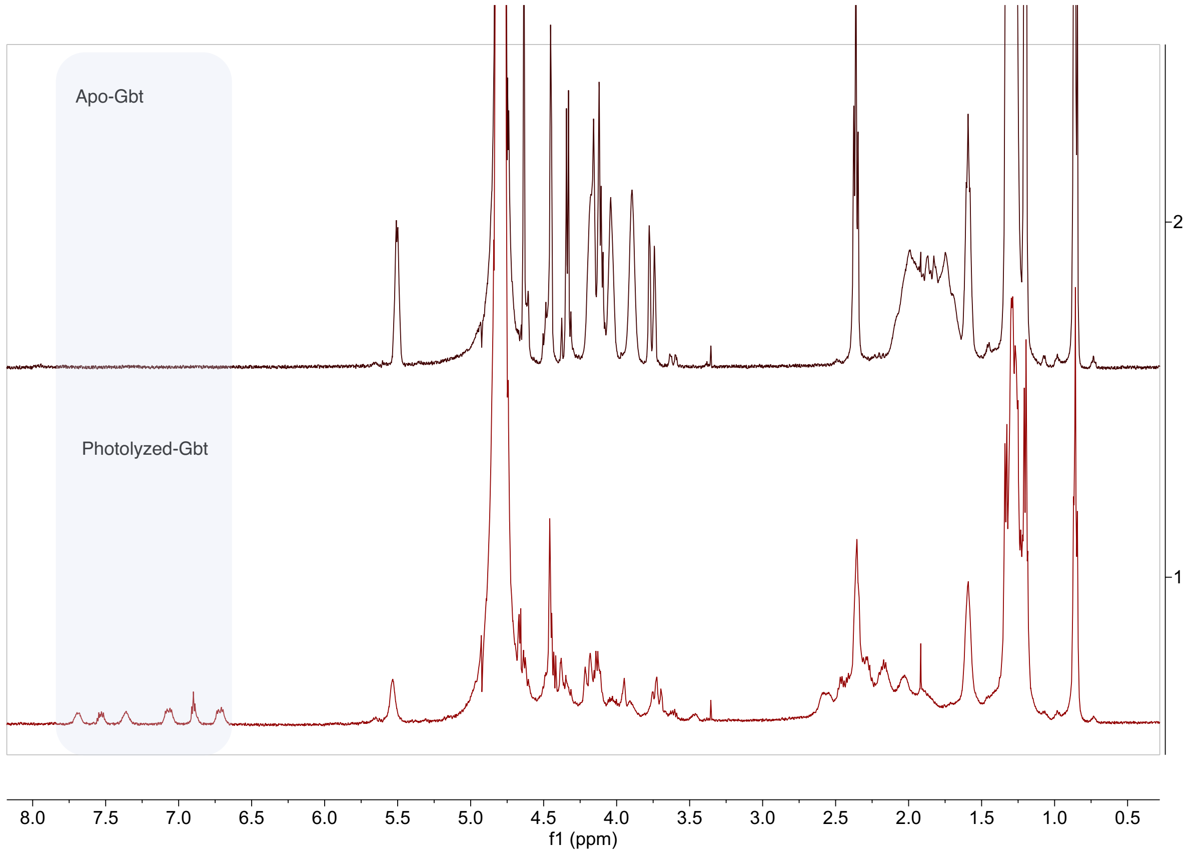

Figure S25. ^{1}H NMR of photolyzed apo-Gbt containing $^{13}C^{15}N$-enriched-Gra. New ^{1}H signals downfield at 6.90 and 7.53 ppm are present which are not observed in apo-Gbt, consistent with the conversion of a diazeniumdiolate to an oxime. Spectra collected on Varian 600 in D_2O.

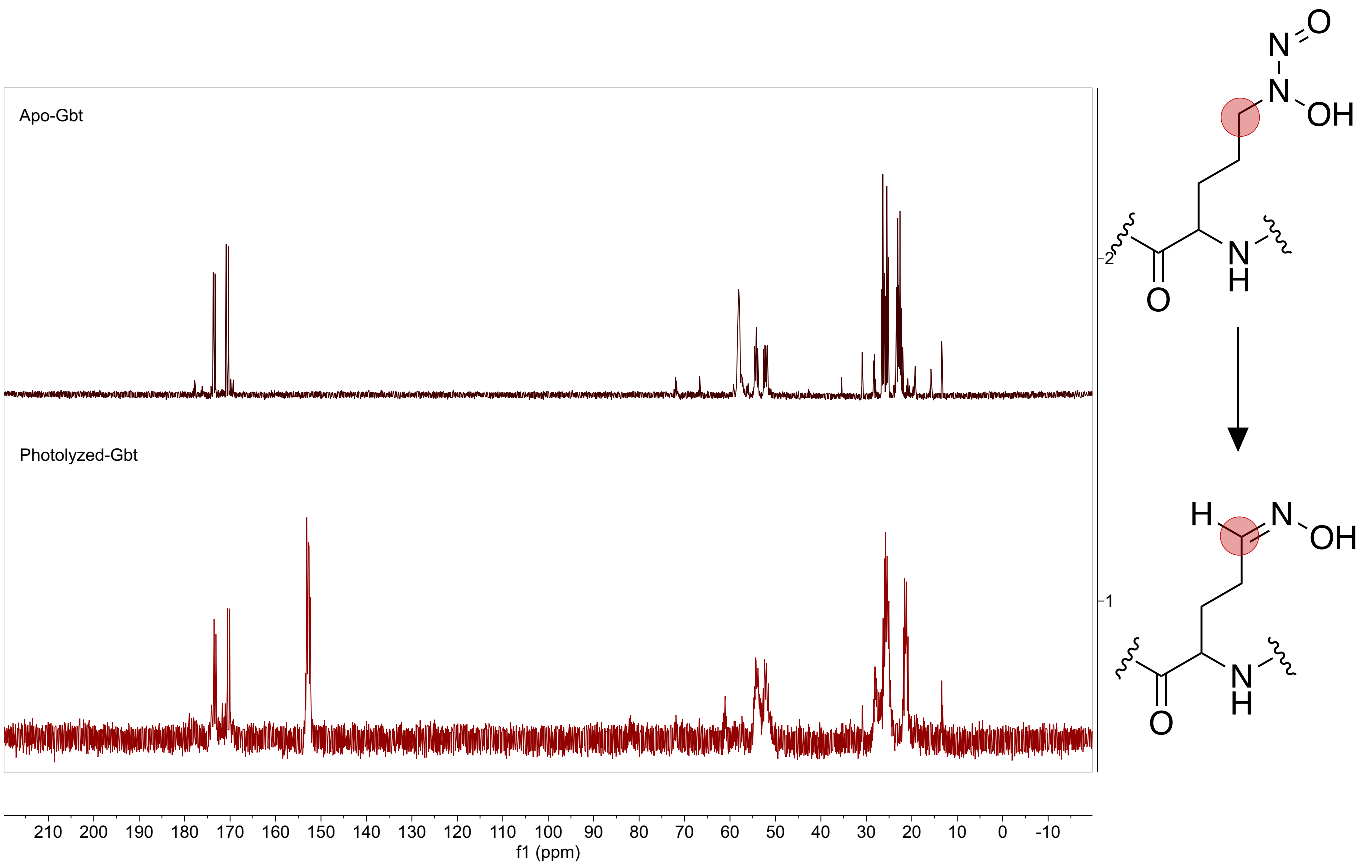

Figure S26. ^{13}C resonances for Cδ on each Gra shift downfield in photolyzed apo-Gbt, consistent with a change from a diazeniumdiolate to an oxime. Data collected on Bruker 500 in D_2O.

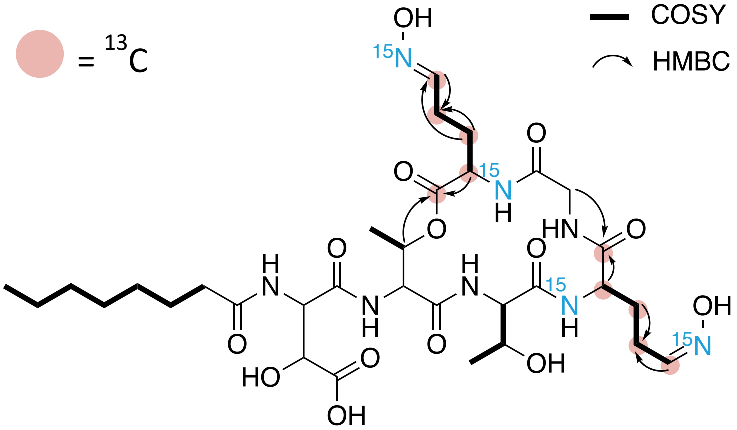

Figure S27. Schematic summary showing COSY and HMBC correlations in photolyzed-^{13}C^{15}N-Gra-enriched-Gbt. Changes were only observed in the Gra residues in photolyzed apo-Gbt.

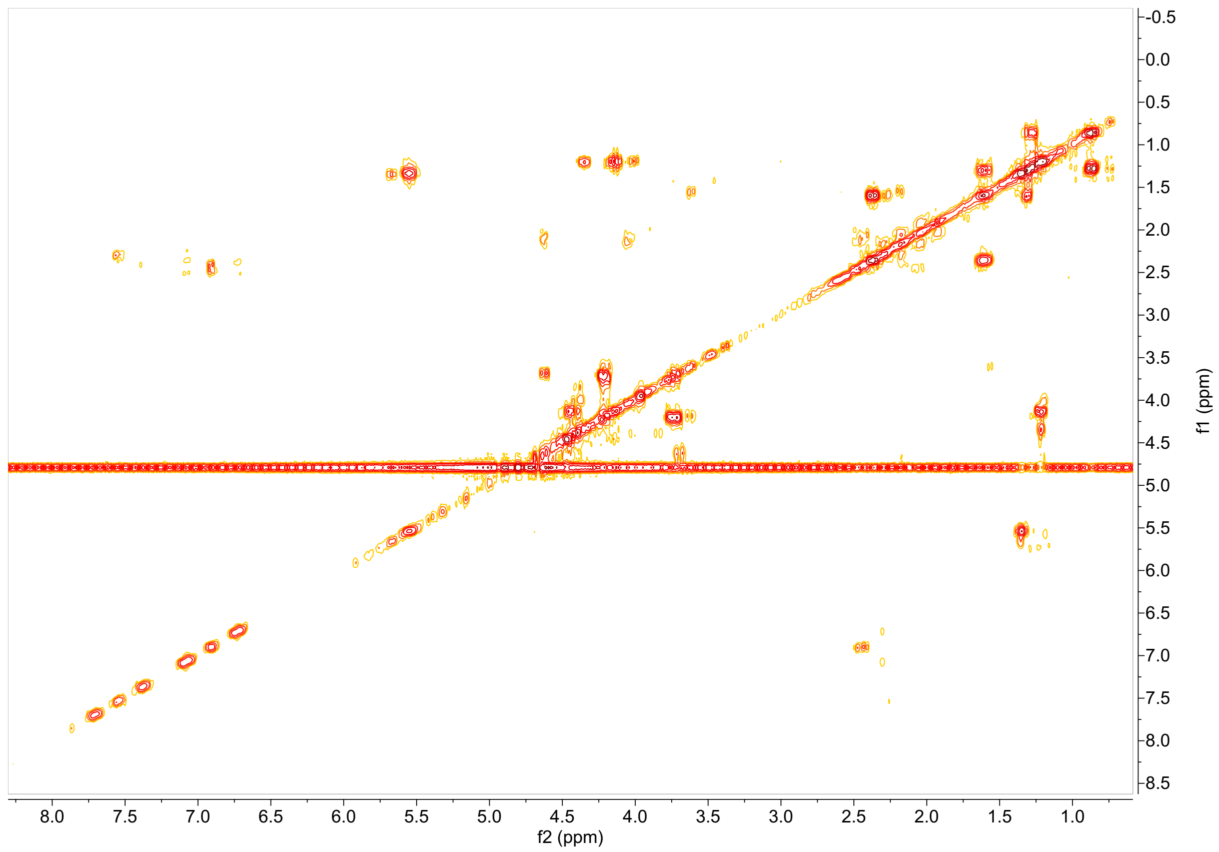

Figure S28. COSY NMR spectrum of photolyzed Gbt shows ^{1}H signals for Cα, Cβ, and Cγ of Gra1 and Gra2 are unchanged after photolysis. The other amino acids in Gbt also remain unchanged. Spectrum collected on Bruker 500 in D_2O.

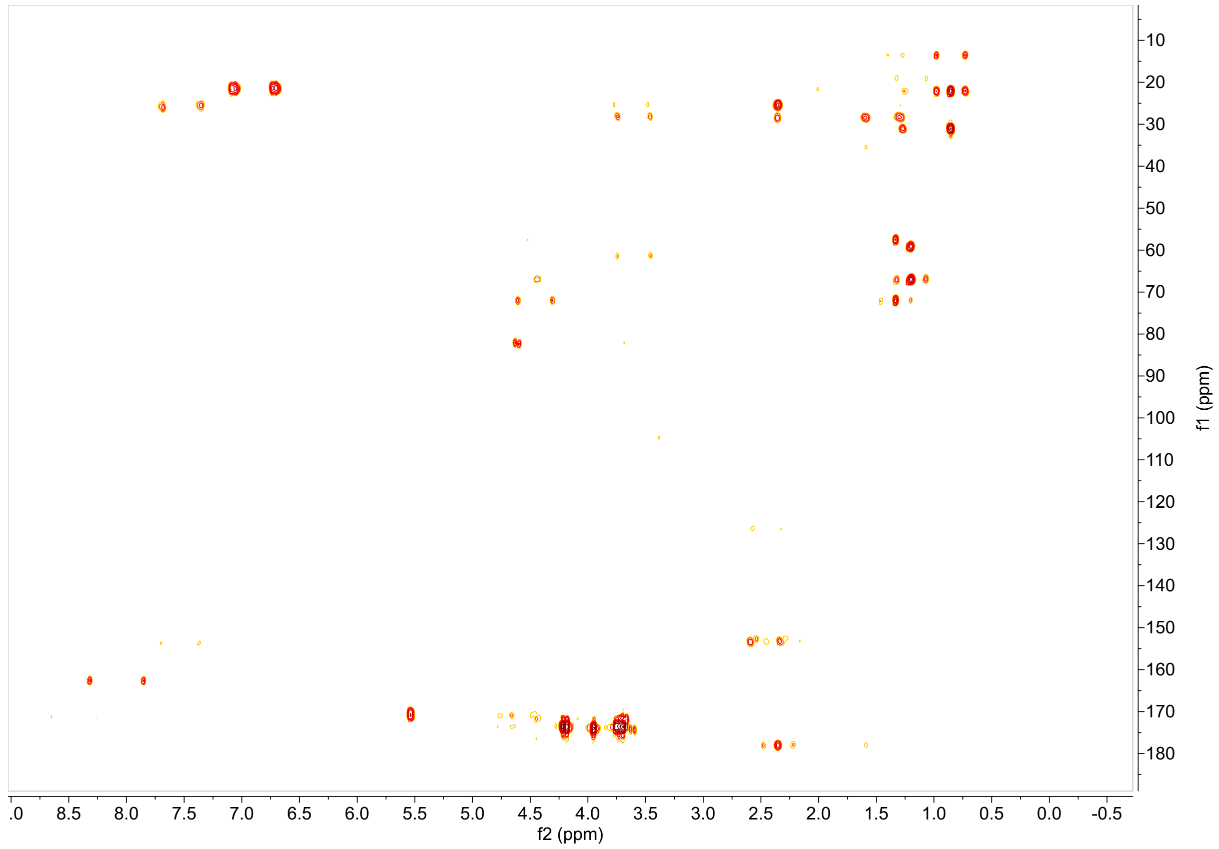

Figure S29. ^{1}H-^{13}C HMBC (with ^{13}C decoupling during acquisition) spectrum of photolyzed $^{13}C^{15}N$-Gra-enriched-Gbt shows ^{1}H and ^{13}C correlations for Cα, Cβ, and Cγ of Gra1 and Gra2 are unchanged after photolysis. The other amino acids in Gbt also remain unchanged. Cδ in photolyzed-$^{13}C^{15}N$-Gra1 and Gra2 show HMBC correlation to Cγ in Gra1 and Gra2. Spectrum acquired on Bruker 500 in D_2O.

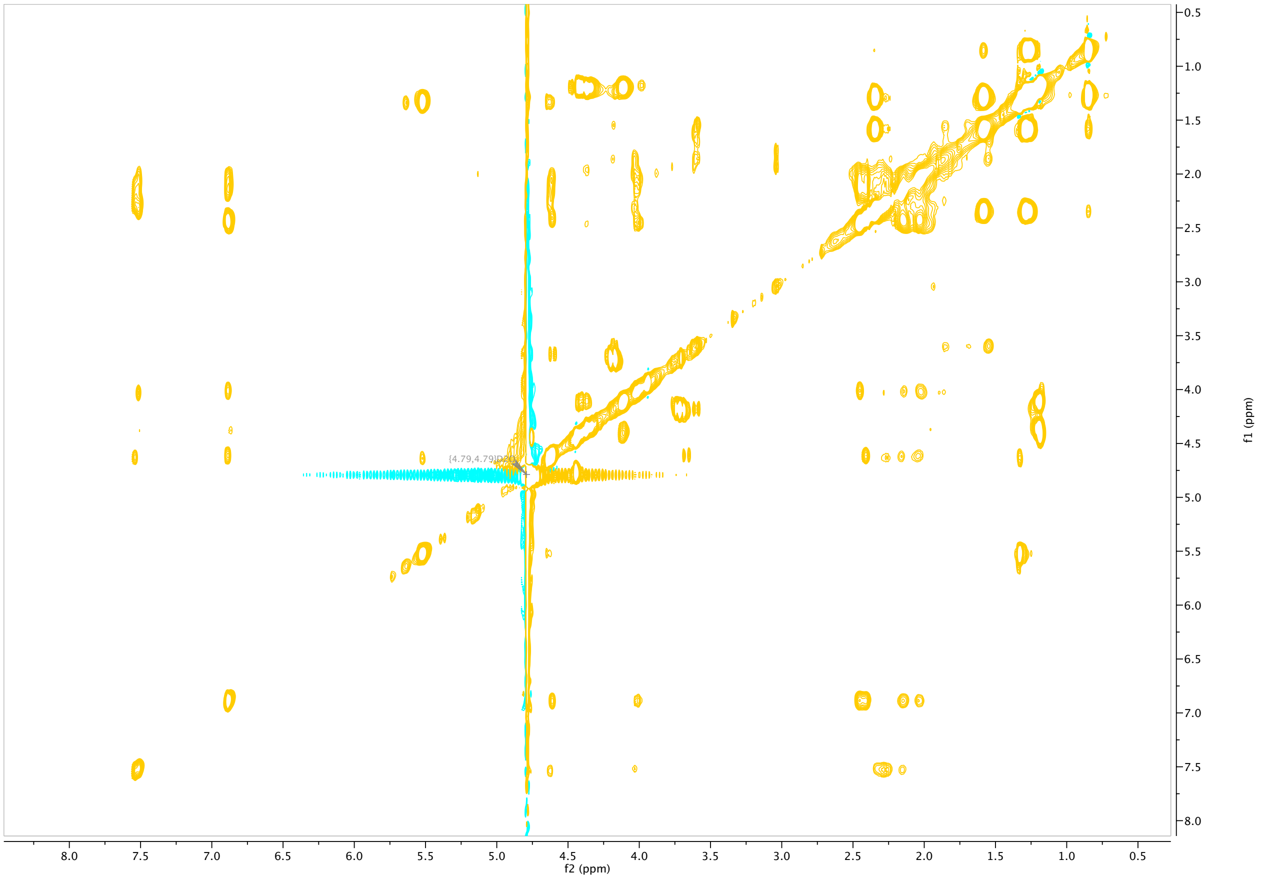

Figure S30. TOCSY spectrum of photolyzed Gbt shows E and Z oxime protons in the total ^{1}H spin systems of both Gra1 and Gra2, but are distinct conformations as can be observed vertically at 6.9 (Z) and 7.5 (E) ppm. Spectrum acquired on Bruker 500 in D_2O.

Table S1. NMR chemical shift table for apo-$^{13}C^{15}N$-Gra-enriched-Gbt in D_2O.

	Residue	Position	^{13}C (ppm)	^{1}H (ppm)	^{13}C-^{13}C COSY	COSY	HSQC (^{1}H, ^{13}C ppm)
1	OH-Asp	NH					
2		CO					
3		Cα		5.00		4	5.00, 56.29 (CH)
4		Cβ		4.71		3	4.72, 70.64 (CH)
5		COOH					
6	Thr1	NH					
7		CO					
8		Cα		4.64			4.64, 57.41 (CH)
9		Cβ	71.80	5.50		10	5.49, 71.77 (CH)
10		Cγ	15.65	1.33		9	1.33, 15.64 (CH_3)
11	Thr2	NH					
12		CO					
13		Cα		4.39		14	4.39, 58.90 (CH)
14		Cβ		4.11		13	4.10, 66.61 (CH)
15		Cγ	18.87	1.19		14	1.19, 18.86 (CH_3)
16	Gra1	NH					
17		CO	173.30		18		
18		Cα	53.91	4.06	17, 19	19	4.06, 53.87 (CH)
19		Cβ	25.36	1.87	18, 20		1.87, 25.36 (CH_2)
20		Cγ	22.91	1.95	21, 19		1.95, 22.91(CH_2)
21		Cδ	61.66	4.20	20		4.20, 61.60 (CH_2)
22	Gly	NH					
23		CO					
24		Cα		3.69, 4.17		24	3.68, 4.17: 42.71 (CH_2)
25	Gra2	NH					
26		CO	170.24		27		
27		Cα	52.11	4.61	26, 28		4.61, 52.06 (CH)
28		Cβ	26.16	1.93	27, 29	29	1.93, 26.16 (CH_2)
29		Cγ	22.26	2.02	30, 28	28	2.02, 22.26 (CH_2)
30		Cδ	61.93	4.22	29	29	4.21, 62.03 (CH_2)
31	OA	CO					
32		Cα		2.36		33	2.35, 35.32 (CH_2)
33		Cβ		1.59		32	1.58, 25.15 (CH_2)
34		Cγ,δ,ε,ζ	28.12, 30.87	1.26		35	1.24: 21.77, 30.96<br>1.28: 25.48, 28.06 (CH_2)
35		Cω	13.29	0.85		34	0.85, 13.22 (CH_3)

Table S2. NMR chemical shifts for apo-$^{13}C^{15}N$-Gra-enriched Gbt in DMSO-d_6.‡

	Residue	Position	^{13}C (ppm)†	^{1}H (ppm)	HSQC (^{1}H, ^{13}C ppm)
1	OH-Asp	NH		8.07 (d)	
2		CO			
3		Cα		4.81 (dd)	4.81, 55.57 (CH)
4		Cβ		4.47	4.47, 70.44 (CH)
5		COOH			
6	Thr1	NH		7.93 (d)	
7		CO			
8		Cα		4.46	4.46, 56.12 (CH)
9		Cβ		5.21	5.21, 70.57 (CH)
10		Cγ	15.93	1.13 (d)	1.13, 15.54 (CH_3)
11	Thr2	NH		7.23 (d)	
12		CO			
13		Cα		4.32 (t)	4.31, 58.23 (CH)
14		Cβ		3.94	3.94, 66.22 (CH)
15		Cγ	19.60	1.02 (d)	1.02, 19.24 (CH_3)
16	Gra1	NH		8.32	
17		CO	171.18		
18		Cα	53.17	3.86	3.86, 52.85 (CH)
19		Cβ	26.20	1.65	1.66, 26.00 (CH_2)
20		Cγ	23.04	1.78	1.78: 22.92, 60.72 (CH_2)
21		Cδ	60.78	4.07	4.06, 60.53 (CH_2)
22	Gly	NH		8.42	
23		CO			
24		Cα		3.35, 4.02 (dd)	3.35, 4.03: 42.29 (CH_2)
25	Gra2	NH		7.56	
26		CO	168.64		
27		Cα	51.28	4.43	4.44, 50.97 (CH)
28		Cβ	22.53	1.72 (m)	1.73, 22.32 (CH_2)
29		Cγ	26.90	1.77 (m)	1.77, 26.16 (CH_2)
30		Cδ	61.28	4.08	4.09, 61.06 (CH_2)
31	OA	CO			
32		Cα		2.17	2.17, 34.82 (CH_2)
33		Cβ		1.46 (t)	1.46, 24.99 (CH_2)
34		Cγ,δ,ε,ζ		1.23 (m)	1.23: 21.59, 25.07, 28.26, 30.90 (CH_2)
35		Cω	13.96	0.85 (t)	0.85, 13.54 (CH_3)

‡ In agreement with published ^{1}H NMR data of unenriched Gbt.[14]

† ^{13}C resonances of $^{13}C^{15}N$ enriched-Gra1 and Gra2 are more intense than ^{13}C resonances in amino acids OH-Asp, Thr1, Thr2, Gly, and OA with signals from natural abundant ^{13}C.

Table S3. NMR chemical shifts for $^{13}C^{15}N$-Gra-enriched-Gbt photoproduct in D_2O.

	Residue	Position	Apo-Gbt ^{13}C (ppm)	Apo-Gbt ^{1}H (ppm)	Photolyzed apo-Gbt ^{13}C (ppm)	Photolyzed apo-Gbt ^{1}H (ppm)	Photolyzed apo-Gbt HSQC
1	OH-Asp	NH					
2		CO					
3		Cα		5.00			4.78, 57.22 (CH)
4		Cβ		4.71			4.46, 71.78 (CH)
5		COOH					
6	Thr1	NH					
7		CO					
8		Cα		4.64			4.66, 57.49 (CH)
9		Cβ	71.80	5.50		5.54	5.54, 71.84 (CH)
10		Cγ	15.65	1.33			1.34, 15.82 (CH_3)
11	Thr2	NH					
12		CO					
13		Cα		4.39			4.41, 59.06 (CH)
14		Cβ		4.11			4.14, 66.73 (CH)
15		Cγ	18.87	1.19			1.22, 18.93 (CH_3)
16	Gra1	NH					
17		CO	173.30		173.31		
18		Cα	53.91	4.06	54.15	4.04	4.04, 54.21 (CH)
19		Cβ	25.36	1.87	25.45	2.02	2.03, 25.53 (CH_2)
20		Cγ	22.91	1.95	21.11	2.39	2.39, 21.03(CH_2)
21		Cδ	61.66	4.20	152.72, 153.10	6.90, 7.53	6.90, 152.60, 7.53, 153.78 (CH)
22	Gly	NH					
23		CO					
24		Cα		3.69, 4.17		3.72, 4.20	3.72, 4.20: 42.80 (CH_2)
25	Gra2	NH					
26		CO	170.24		170.33		
27		Cα	52.11	4.61	52.16	4.63	4.62, 52.31 (CH)
28		Cβ	26.16	1.93	26.02	2.17	2.16, 25.84 (CH_2)
29		Cγ	22.26	2.02	21.55	2.45	2.45, 21.51 (CH_2)
30		Cδ	61.93	4.22	152.72, 153.10	6.90, 7.53	6.90, 152.60, 7.53, 153.72(CH)
31	OA	CO					
32		Cα		2.36		2.35	2.36, 35.34 (CH_2)
33		Cβ		1.59			1.59, 25.39 (CH_2)
34		Cγ,δ,ε,ζ	28.12, 30.87	1.26			1.27: 21.87, 25.40, 28.31, 31.05, (CH_2)
35		Cω	13.29	0.85			0.86, 13.47 (CH_3)

References

(1) Ng, T. L.; Rohac, R.; Mitchell, A. J.; Boal, A. K.; Balskus, E. P. An N-nitrosating metalloenzyme constructs the pharmacophore of streptozotocin. *Nature* **2019**, *566* (7742), 94-99. DOI: 10.1038/s41586-019-0894-z.

(2) Romo, A. J.; Shiraishi, T.; Ikeuchi, H.; Lin, G. M.; Geng, Y.; Lee, Y. H.; Liem, P. H.; Ma, T.; Ogasawara, Y.; Shin-Ya, K.; et al. The Amipurimycin and Miharamycin Biosynthetic Gene Clusters: Unraveling the Origins of 2-Aminopurinyl Peptidyl Nucleoside Antibiotics. *J Am Chem Soc* **2019**, *141* (36), 14152-14159. DOI: 10.1021/jacs.9b03021

(3) Kumagai, T.; Takagi, K.; Koyama, Y.; Matoba, Y.; Oda, K.; Noda, M.; Sugiyama, M. Heme protein and hydroxyarginase necessary for biosynthesis of D-cycloserine. *Antimicrob Agents Chemother* **2012**, *56* (7), 3682-3689. DOI: 10.1128/AAC.00614-12.

(4) Chu, L.; Luo, X.; Zhu, T.; Cao, Y.; Zhang, L.; Deng, Z.; Gao, J. Harnessing phosphonate antibiotics argolaphos biosynthesis enables a synthetic biology-based green synthesis of glyphosate. *Nat Commun* **2022**, *13* (1), 1736. DOI: 10.1038/s41467-022-29188-6.

(5) Filgueiras, M. F.; de Jesus, P. C.; Borges, E. M. Quantification of Nitrite in Food and Water Samples Using the Griess Assay and Digital Images Acquired Using a Desktop Scanner. *Journal of Chemical Education* **2021**, *98* (10), 3303-3311. DOI: 10.1021/acs.jchemed.0c01392.

(6) Miranda, K. M.; Espey, M. G.; Wink, D. A. A rapid, simple spectrophotometric method for simultaneous detection of nitrate and nitrite. *Nitric Oxide* **2001**, *5* (1), 62-71. DOI: 10.1006/niox.2000.0319.

(7) Kolluru, G. K.; Yuan, S.; Shen, X.; Kevil, C. G. H2S regulation of nitric oxide metabolism. *Methods Enzymol* **2015**, *554*, 271-297. DOI: 10.1016/bs.mie.2014.11.040.

(8) Kanehisa, M. Toward understanding the origin and evolution of cellular organisms. *Protein Science* **2019**, *28* (11), 1947-1951. DOI: https://doi.org/10.1002/pro.3715.

(9) Kanehisa, M.; Furumichi, M.; Sato, Y.; Ishiguro-Watanabe, M.; Tanabe, M. KEGG: integrating viruses and cellular organisms. *Nucleic Acids Research* **2020**, *49* (D1), D545-D551. DOI: 10.1093/nar/gkaa970.

(10) Kanehisa, M.; Goto, S. KEGG: Kyoto Encyclopedia of Genes and Genomes. *Nucleic Acids Research* **2000**, *28* (1), 27-30. DOI: 10.1093/nar/28.1.27.

(11) J.P. Marchal, D. C. 15N Chemical Shifts and One Bond 15N-1H Coupling Constants in Simple Amides. *Organic Magnetic Resonance* **1981**, *15* (4), 344-346.

(12) Balaz, M.; Kudlickova, Z.; Vilkova, M.; Imrich, J.; Balazova, L.; Daneu, N. Mechanochemical Synthesis and Isomerization of N-Substituted Indole-3-carboxaldehyde Oximes dagger. *Molecules* **2019**, *24* (18). DOI: 10.3390/molecules24183347.

(13) Patteson, J. B.; Putz, A. T.; Tao, L.; Simke, W. C.; Bryant, L. H., 3rd; Britt, R. D.; Li, B. Biosynthesis of fluopsin C, a copper-containing antibiotic from Pseudomonas aeruginosa. *Science* **2021**, *374* (6570), 1005-1009. DOI: 10.1126/science.abj6749.

(14) Hermenau, R.; Ishida, K.; Gama, S.; Hoffmann, B.; Pfeifer-Leeg, M.; Plass, W.; Mohr, J. F.; Wichard, T.; Saluz, H. P.; Hertweck, C. Gramibactin is a bacterial siderophore with a diazeniumdiolate ligand system. *Nat Chem Biol* **2018**, *14* (9), 841-843. DOI: 10.1038/s41589-018-0101-9.
